# Late-Onset Behavioral and Synaptic Consequences of L-Type Ca^2+^ Channel Activation in the Basolateral Amygdala of Developing Rats

**DOI:** 10.1523/ENEURO.0282-21.2022

**Published:** 2022-02-22

**Authors:** Yiming Zhang, Anne-Sophie Sack, Karen L. Jones, Yi Yang, Esperanza Garcia, Terrance P. Snutch

**Affiliations:** 1 Michael Smith Laboratories, University of British Columbia, Vancouver, British Columbia V6T 1Z4, Canada; 2 Djavad Mowafaghian Centre for Brain Health, University of British Columbia, Vancouver, British Columbia V6T 1Z3, Canada

**Keywords:** amygdala, cell excitability, L-type calcium channel, synaptic plasticity

## Abstract

Postnatal CNS development is fine-tuned to drive the functional needs of succeeding life stages; accordingly, the emergence of sensory and motor functions, behavioral patterns and cognitive abilities relies on a complex interplay of signaling pathways. Strictly regulated Ca^2+^ signaling mediated by L-type channels (LTCCs) is crucial in neural circuit development and aberrant increases in neuronal LTCC activity are linked to neurodevelopmental and psychiatric disorders. In the amygdala, a brain region that integrates signals associated with aversive and rewarding stimuli, LTCCs contribute to NMDA-independent long-term potentiation (LTP) and are required for the consolidation and extinction of fear memory. *In vitro* studies have elucidated distinct electrophysiological and synaptic properties characterizing the transition from immature to functionally mature basolateral subdivision of the amygdala (BLA) principal neurons. Further, acute increase of LTCC activity selectively regulates excitability and spontaneous synaptic activity in immature BLA neurons, suggesting an age-dependent regulation of BLA circuitry by LTCCs. This study aimed to elucidate whether early life alterations in LTCC activity subsequently affect synaptic strength and amygdala-dependent behaviors in early adulthood. *In vivo* intra-amygdala injection of an LTCC agonist at a critical period of postnatal neurodevelopment in male rat pups was used to examine synaptic plasticity of BLA excitatory inputs, expression of immediate early genes (IEGs) and glutamate receptors, as well as anxiety and social affiliation behaviors at a juvenile age. Results indicate that enhanced LTCC activity in immature BLA principal neurons trigger persistent changes in the developmental trajectory to modify membrane properties and synaptic LTP at later stages, concomitant with alterations in amygdala-related behavioral patterns.

## Significance Statement

Early-life alterations to normal developmental processes are known to change the morphology and functioning of the amygdala in the adult brain and are correlated to emotional and behavioral disorders. However, the precise cellular and molecular determinants of key neurodevelopmental stages predisposing the amygdala circuitry to pathologic states remain to be fully defined. Our study provides evidence that Ca^2+^ signaling through L-type calcium channels (LTCCs) during a vulnerable period of postnatal development is linked to long-lasting synaptic remodeling associated to altered behavioral outcomes in early adulthood. The results expand our knowledge concerning the possible pathophysiologies underlying psychiatric disorders of neurodevelopmental origin.

## Introduction

Early brain development involves the integration of numerous cellular and molecular mechanisms that must occur with temporal and spatial precision (Dehorter et al., 2012). Calcium signaling through neuronal L-type calcium channels (LTCCs) Ca_v_1.2 and Ca_v_1.3 critically influences neurite outgrowth, myelination, synaptogenesis and fine-tuning of neuronal networks (Cheli et al., 2016; Oh et al., 2016; Kamijo et al., 2018). At the molecular level, Ca^2+^ entry through LTCCs initiates signal transduction cascades that activate immediate early gene (IEG) transcription and regulate transcription factors during development (West and Greenberg, 2011; Wild et al., 2019; Perfitt et al., 2020).

The crucial roles of LTCCs in regulating neural circuit maturation is further evident from cognitive and behavioral disorders linked to LTCC gene dysfunction. For example, Timothy syndrome (TS), is a severe disorder associated with core symptoms of autism spectrum disorder (ASD) and results from gain-of-function mutations in the Ca_v_1.2 and Ca_v_1.3 LTCC genes (Splawski et al., 2005). Further, polymorphisms and loss-of-function LTCC variants are linked with autism and schizophrenia (Dedic et al., 2018; Kisko et al., 2018; Moon et al., 2018).

Neuroimaging studies in humans and experimental evidence in rodents have revealed a key involvement of the amygdala in neurodevelopmental disorders (Benarroch, 2015; Herrington et al., 2017). In animal models of ASD, functional alterations of the basolateral subdivision of the amygdala (BLA) result in the impairment of social interactions (Truitt et al., 2007; Sosa-Díaz et al., 2014). Further, experience-dependent synaptic plasticity in the amygdala underlies fear processing, anxiety and social activities (Rainnie et al., 2004; Truitt et al., 2007).

Seminal studies of the postnatal maturation of rat BLA neurons show substantial changes in resting membrane potential, input resistance, firing patterns and synaptic activity occurring between postnatal day P7 and P21, concomitant with the expansion of dendritic arbor (Ehrlich et al., 2009, 2012, 2013; Ehrlich and Rainnie, 2015; Ryan et al., 2016). These studies have led to the notion that the first three weeks of rat postnatal life represent a critical vulnerable period and that pathologic alterations can cause permanent changes in the BLA circuitry, in line with the hypotheses of developmental etiology of ASD and schizophrenia (Meredith, 2015).

Prenatal stress can induce age-specific changes in socioemotional behavior in rats through alterations in BLA excitability, suggesting that developmental manipulation of membrane electrical properties can produce long-lasting changes in the amygdala network (Ehrlich and Rainnie, 2015). Our recent study employing rat brain slices showed that acute application of the LTCC agonist (S)-BayK8644 (BayK) evoked a robust increase in action potential firing and an overall increase in BLA excitability with significantly higher impact on P7 immature neurons compared with P21 neurons (Zhang et al., 2020). However, it remains to be elucidated whether LTCC-mediated BLA hyperexcitability at P7 could subsequently translate into physiologically relevant changes in amygdala-dependent behaviors in early adulthood.

To examine longer-term effects of increased LTCC activity, stereotaxic bilateral injections of BayK into the BLA were performed in rat pups at P7, P14, or P21. Electrophysiological, synaptic and behavioral characteristics were examined at P28, a juvenile stage when the BLA circuit has reached maturity. BayK injection was found associated with noted changes in neuronal membrane properties, long-term potentiation (LTP) of excitatory inputs, ionotropic glutamate receptor subunit expression, impairment of social interaction ability, increased anxiety and the presence of repetitive behaviors. Together, the results support the hypothesis that the BLA network is prone to functional manipulation of LTCC activity, and provide evidence of a key role of LTCCs in regulating the developmental organization of mature networks sustaining complex behaviors.

## Materials and Methods

### Animals

All experimental protocols were approved by the Animal Care Committee (ACC protocol A16-0127) of the University of British Columbia (UBC). Pregnant Sprague Dawley rats from Charles River Laboratories (17 d of pregnancy on the day of arrival) were housed at room temperature (24–26°C) in a 12/12 h light/dark cycle at UBC Animal Resource Unit. To minimize any potential effects of environmental changes we followed the UBC SOP on handing of rat and mouse pups that undergo procedures (September 27, 2016) for habituation. Briefly, rat pups were handled initially in the presence of the mother and then separated from the mother (30 min/d) from day 0 to the day of surgery. Rat pups were weaned at P21. Pups were randomly assigned to three experimental groups with each of the groups receiving a one-time BayK or DMSO bilateral injection at a specific age (P7, or P14, or P21). Male rats were used in all experiments. While the present study was limited to sampling males, we expect that future studies involving females may identify and consider any important sex differences relevant to BLA postnatal development and LTCC involvement (Guadagno et al., 2020).

### Stereotaxic surgery

The stereotaxic injection of vehicle (DMSO) or BayK was performed on rat male pups from different litters at P7, P14, or P21 using a stereotaxic frame (David Kopf Instruments, Model 900) with a resolution of 100 μm. Initially, rat pups were placed in an acrylic chamber and anesthetized with isoflurane using a VetEquip vaporizer; anesthesia was maintained throughout the surgery using a nose cone/face mask. Once the surgical plane of anesthesia was reached, the skin over the cerebrum was incised by a 1 cm-long median-sagittal cut. Two symmetrical holes were made using a dental drill on each side of the skull over the cerebrum (P7: bregma −2.4 mm anterior-posterior, ±3.4 mm medial-lateral, 6.8 mm dorsal-ventral; P14: bregma −2.6 mm anterior-posterior, ±3.8 mm medial-lateral, 7 mm dorsal-ventral; P21: bregma −3.8 mm anterior-posterior, ±4.0 mm medial lateral, 7.2 mm dorsal-ventral). Bregma coordinates were established for each age considering the rapid change in the position of brain structures during the periods examined (Khazipov et al., 2015). A Hamilton Neuros micro-syringe was inserted on each side to deliver 0.5 μl of a 1 mm BayK solution (corresponding to a calculated dose of 8 μg/kg) or the same volume of DMSO vehicle into the BLA regions. The injection duration was 5 min at a rate of 0.1 μl/min. After injection, the microsyringe needle was left stationary for a further 3 min before retrieval. After completion of the procedures, the skin was sutured with subcuticular sutures (6–0 coated Vicryl). To attenuate surgical pain in pups, bupivacaine (diluted in saline solution to 0.25%) was dripped onto the skin when closing the incision at the end of surgery.

In order to verify the location of bilateral injection within the BLA, a subset of naive rats received 0.2 μl methylene blue (20%) stereotaxic injection at either P7, P14, or P21. Animals were euthanized, brains dissected and 300 μm slices collected with a vibratome (VT 1200, Leica) and visually examined under the microscope. The area of BLA containing methylene blue confirmed the accuracy and consistency of the bilateral injection. In addition, to verify the diffusion area of BayK injection in BLA, animals (*n* = 4) were euthanized, brains dissected 1 h after BayK injection, slices prepared and observed under the microscope. The BLA area showed a rounded area of ∼1 mm in diameter which was visible because of the yellow color of the BayK solution.

### Acute brain slice preparation

At age P28, animals were anesthetized using isoflurane [5% in oxygen (v/v)] and decapitated using a rodent guillotine. The brain was quickly removed and transferred to ice-cold sucrose cutting solution: 214 mm sucrose, 26 mm NaHCO_3_, 1.25 mm NaH_2_PO_4_, 11 mm glucose, 2.5 mm KCl, 0.5 mm CaCl_2_, and 6 mm MgCl_2_, bubbled constantly with 95% O_2_/5% CO_2_. Trimmed brain tissue was glued to the cutting chamber of a vibratome. Coronal brain slices containing amygdala were cut to 300 μm thickness then incubated at 32°C for 1 h in artificial cerebrospinal fluid (ACSF): 130 mm NaCl, 30 mm NaHCO_3_, 3.5 mm KCl, 1.1 mm KH_2_PO_4_, 1.3 mm MgCl_2_, 2.5 mm CaCl_2_, 10 mm glucose, 0.4 mm sodium ascorbate, 0.8 mm thiourea, and 2 mm sodium pyruvate, saturated with 95% O_2_/5% CO_2_.

### Electrophysiological recordings

Acute brain slices were transferred to a recording chamber (234 μl total volume; Warner RC-26G) mounted on the stage of an upright microscope (Zeiss Axioskop 2) and maintained in ACSF perfusion at a flow rate of 2 ml/min. An Ag/AgCl pellet was used for grounding the recording chamber.

BLA pyramidal neurons were visually identified using infrared differential interference contrast (IR-DIC) in combination with a 40× water immersion objective. To confirm the localization of the BLA area, a subset of BLA neurons were labeled with biocytin (0.05% in internal pipette solution). All electrophysiological recordings were performed using a Multiclamp 700B amplifier with pClamp software version 11 and digitized with a DigiData 1550B (Molecular Devices) acquisition system.

Whole-cell patch-clamp recordings were performed in standard ACSF solution. An AP-1000 micropipette puller (Sutter Instrument) was used to pull patch pipettes from thick wall borosilicate capillary glass with final resistances of 3–5 MΩ. For neuronal action potential recordings in current clamp, pipettes were filled with the following internal solution: 130 mm K gluconate, 2 mm KCl, 10 mm HEPES, 3 mm MgCl_2_, 2 mm K-ATP, 0.2 mm Na-GTP, and 5 mm phosphocreatine Tris, the pH was adjusted to 7.3 with KOH, and osmolality was adjusted to 290 mOsmol/L with D-Mannitol. Cells with a resting membrane potential less than −55 mV, or access resistance >30 MΩ, or action potentials exhibiting no overshoot were excluded. The access resistance and bridge balance values were checked throughout the recording and cells with >15% changes were not included in data analyses. Current-clamp values are reported without subtracting the liquid junction potential, calculated as 11.4 mV. For current–voltage (*I-V*) relationship analyses, steady-state voltage changes were measured and averaged over a 400-ms period at the end of a 1 s square current pulse, and the input resistance (R_in_) calculated from the linear part of the *I-V* curve near the resting membrane potential. The steady-state frequency of action potentials was obtained from the last 400 ms of the depolarizing pulses, and plotted as the function of normalized current injection for frequency-current (*f-I*) relationship. Gain was measured as the slope of the initial linear part of *f-I* curve. The recordings were obtained and low-pass filtered at 10 kHz and digitized at 50 kHz.

The following internal solution was used to record evoked EPSCs: 134.63 mm Cs-methanesulfonate, 5 mm CsCl, 5 mm TEA-Cl, 0.4 mm EGTA, 10 mm HEPES, 2.5 mm Mg-ATP, 2.5 mm Na-GTP, and 5 mm phosphocreatine Tris; the pH was adjusted to 7.3 with CsOH and osmolality was adjusted to 290 mOsm/L with D-Mannitol. To examine evoked EPSCs, BLA cells were held at −60 mV and a concentric bipolar stimulating electrode (CBAPC100 from FHC Inc.) was placed on the lateral amygdala (LA). Single pulses were generated with an S48 Stimulator via a Stimulus Isolation Unit SIU5 (Grass Instruments) at 0.05 Hz. In order to induce LTP, high-frequency stimulation (HFS) was applied at 100 Hz for 1 s, repeated five times with an interval of 10 s, while the cells were held at a membrane potential of +30 mV. EPSCs were recorded in ACSF containing GABA_A_ receptor antagonist picrotoxin (PTX 100 μm) and GABA_B_ receptor antagonist CGP52432 (1 μm). The amplitude of EPSCs was calculated as the peak current in the first 50 ms following the stimulation artifact. Data acquisition was sampled at 2 kHz and filtered at 50 kHz.

Two different approaches were used to examine the effect of BayK on synaptic plasticity: in one group of animals the induction of LTP was tested subsequent to the behavioral tests; in a second group, LTP recordings were performed without animals having been exposed to any prior behavioral experience.

### Behavioral tests

All tests were performed during the dark period of the day/night cycle in a quiet symmetrically designed test room under dark conditions. Animal behavior was recorded with an overhead infrared camera (Amcrest ProHD 1080P) placed 1.5 m away above the test chamber. At P28 the juvenile animals were examined in the following ASD-related behavioral tests:

#### Three chamber test

Rodents have a preference to spend time with conspecifics and this test is used to assess cognition in the form of general sociability and interest in social novelty in rodent models of CNS disorders (Malkova et al., 2012). Experimental animals were tested in a 0.89 m L × 0.48 m W × 0.4 m H Plexiglas box divided into three chambers; rats could freely move between chambers through a small opening (10 × 8 cm). At the beginning of the session, rats were placed in the central chamber for 10 min to explore the empty box to evaluate bias for either of the side chambers. Once we confirmed that the animal had no evident bias, a stranger rat of the same sex and similar body weight was placed in a wire cage (12 cm in height, 9 cm in diameter) on one lateral chamber chosen randomly; a second, identical empty wire cage was placed on the opposite side chamber as a nonsocial object. The time spent in each of the three chambers was measured and social preference calculated as [time_social chamber_/(time_social chamber_ + time_nonsocial chamber_)] × 100 – 50. The test chambers were cleaned with 70% ethanol at the end of each session.

#### Social interaction test

Social interaction behaviors are employed in juvenile and adult rats for evaluating social motivation and nonplay social behaviors (Vanderschuren et al., 2016; Pellis et al., 2018). Experimental animals were placed individually in the test box (0.89 m L × 0.48 m W × 0.4 m H Plexiglas box with an open-top) 10 min before the test for habituation. Then, an age-matched, sex-matched, and weight-matched stranger rat, which had not been housed together before the test, was placed in the test box. Social interaction behaviors of the two juvenile rats were recorded for 10 min. The frequency and duration of the following behavioral patterns from experimental animals were measured with a chronometer by a blind-to-treatment observer using the video recording: (1) chasing/sniffing, the experimental rat follows the stranger rat and sniffs its back and genital area; this parameter evaluates social exploration; (2) climbing, a dominant posture in which the experimental rat stands over the back or the exposed ventral area of the stranger rat and with its front paws presses the shoulder of the opponent against the floor; this parameter evaluates the ability to engage in active social interaction with conspecifics.

#### Self-grooming test

Self-grooming can provide an index of repetitive/stereotypic behavior in rodents (Silverman et al., 2010). Individual rats were placed into an inverted glass beaker of 10-cm diameter × 15-cm height, covered with a filter top, for 10 min as it is known that a restricted environment induces repetitive behavior (Lewis et al., 2007). Self-grooming behavior was then evaluated by recording the number and duration of self-grooming for a further 10 min. All beakers were thoroughly cleaned with 70% ethanol before and after each test.

#### Open field test

This test was originally developed to assess for emotionality or locomotor activity in rodents; more recently it has been widely used for testing the level of anxiety. The animal was placed into the open field (0.4 m L × 0.32 m W × 0.3 m H Plexiglas box) and behaviors recorded over? 10 min. Rodents typically prefer not to be in the center, and tend to walk close to the walls since the chamber is a novel, presumably stressful, environment. A decreased amount of time spent in the center area (0.18 m L × 0.14 m W) of the open field may indicate a higher anxiety level.

### Quantitative real-time PCR (qPCR)

BLA tissue samples were microdissected from 300 μm coronal brain sections using a stainless-steel brain punch tissue set (Leica Biosystems) to examine mRNA expression levels of IEGs and ionotropic glutamatergic receptors using qPCR (Palkovits, 1985). For IEGs *c-fos*, *arc*, and *Homer1a*, tissue samples were collected at 1, 3, or 12 h after stereotaxic injection of BayK or DMSO. For ionotropic glutamatergic receptors GluA and GluN genes, tissue samples were collected at P28 (Table 1). BLA samples were either stored at −80°C or processed immediately. Tissue was homogenized and total RNA was isolated using Dynabeads Magnetic Beads (MicroMod) and MagMax 96 for Microarrays Total RNA Isolation kit (Ambion), then dissolved in TE buffer (IDT). RNA was reverse transcribed into first-strand cDNA with the High-Capacity DNA Reverse Transcription kit (Thermo Fisher Scientific) under the following conditions in a Bio-Rad Thermal cycler: 25°C for 10 min, 37°C for 120 min, and 85°C for 5 s. All qPCR probes were obtained from Integrated DNA Technologies (IDT); primer sequences are given in Table 1. cDNA samples and probes were prepared with KAPA Probe Fast 2× Mix (Sigma) and loaded as triplicates into a 384-well processing plate. Real-time qPCR was performed in a Bio-Rad CFX384 Touch Real-Time PCR system with an initial step of 98°C for 2 min, then the cycling conditions at 98°C for 5 s, 57.5°C for 15 s, with 40 cycles total, followed by finishing steps at 56.6°C for 5 s and 98°C for 50 s. All samples were processed in triplicate and expression levels of each product were normalized to the expression of GAPDH. Ct values were measured from the regression function of the amplification curve. Data are reported as mean ± SEM. Statistical comparison was performed with one-way ANOVA with Bonferroni test.

**Table 1:** Real-time qPCR probe sequences

Genes	Gene ID	Probe	Primer 1	Primer 2
C-fos	314322	5′-/56-FAM/CTGTCAACA/ZEN/CACAGGACTTTTGCGC /3IABkFQ/−3′	5′-CAGCCTTTCCTACTACCATTCC-3′	5′-TTGGCACTAGAGACGGACA-3′
Arc	54323	5′-/56-FAM/CCCCCAGCA/ZEN/GTGATTCATACCAGT /3IABkFQ/−3′	5′-TGAAGCAGCAGACCTGAC-3′	5′-GAGTCATGGAGCCGAAGTC-3′
Homer1	29546	5′-/56-FAM/TCCATCTTC/ZEN/TCCTGCGACTTCTCCT /3IABkFQ/−3′	5′-TCTCCTCTGAGCATCATCTCTC-3′	5′-CTCCTGCTGATTCCTGTGAAG-3′
GriA1	50592	5′-/56-FAM/AGAAGCCAC/ZEN/AGAAGTCCAAGCCA /3IABkFQ/−3′	5′-TTCTCCAAGCCATTCATGAGT-3′	5′-CGACGCTCACTCCAATGTAG-3′
GriA2	29627	5′-/56-FAM/TCACCAATG/ZEN/CTTTCTGCTCCCAGT /3IABkFQ/−3′	5′-CTGACACCCCATATCGACAA-3′	5′-TCCAAAAATTGCGTAGACTCCT-3′
GriA3	29628	5′-/56-FAM/TGTGACTAA/ZEN/TGCTTTCTGCTCCCAGTT /3IABkFQ/−3′	5′-GCCCTTCCATTTGAATTACCAC-3′	5′-AAAGATAGCATACACCCCTCTG-3′
Grin1	24408	5′-/56-FAM/CGCCTACTC/ZEN/CCAACGACCACTTC /3IABkFQ/−3′	5′-GATTCTGTAGAAGCCAGCTGT-3′	5′-TCATCTCTAGCCAGGTCTACG-3′
Grin2A	24409	5′-/56-FAM/CGTCCAACT/ZEN/TCCCGGTTTTCAAGC /3IABkFQ/−3′	5′-ACCAGTTTACAGCCTTCATCC-3′	5′-GCACCAGTACATGACCAGATTC-3′
Grin2B	24410	5′-/56-FAM/TCTGCCTTC/ZEN/TTAGAGCCATTCAGCG /3IABkFQ/−3′	5′-CACAAACATCATCACCCACAC-3′	5′-GCATCAGTGTCATGGTATCTCG-3′
GAPDH	24383	5′-/56-FAM/CACACCGAC/ZEN/CTTCACCATCTTGTCT /3IABkFQ/−3′	5′-TCTCTGCTCCTCCCTGTTC-3′	5′-GTAACCAGGCGTCCGATAC-3′

The table lists IUPHAR (International Union of Basic and Clinical Pharmacology Committee) gene names, GenBank accession number (gene ID), and RT-qPCR probe and primer oligonucleotide sequences provided by Integrated DNA Technologies (IDT).

### Data analysis

Electrophysiological data analyses were performed using Clampfit version 11 (Molecular Devices) and Origin version 2019 (OriginLab). Data are reported as mean ± SEM. Statistical comparison was performed using hypothesis testing (unpaired Student’s *t* test; Welch correction where appropriate). Student’s *t* test was used to analyze the significance between BayK and DMSO treatment groups in behavioral tests. A *p* value < 0.05 was considered to indicate statistical significance. Estimation statistics, which provide quantitative answers and information about the precision of each measurement by using effect size and confidence intervals (CIs; Gardner and Altman, 1986; Ho et al., 2019; Bernard, 2019; Calin-Jageman and Cumming, 2019), was also used to analyze the data where appropriate using GraphPad Prism 9 (GraphPad Software). In the estimation plots in Figures 2, 3, the left axis is used for the scatter graph where individual data points are plotted, the right axis shows the difference between means with a 95% CI (in blue); dotted lines are aligned to each respective mean. In Figures 4, 6, the line in the box plot represents the median, the open square represents the mean value, and whiskers are set to cover the minimum and maximum of the data values (Krzywinski and Altman, 2014). All *n* values reported as sample size represents number of neurons recorded in electrophysiology experiments, or number of animals in behavioral tests and real-time qPCR experiments.

### Drugs

(S)-Bay K8644, nifedipine, D-AP5, CNQX, CGP52432, and QX-314 were purchased from Tocris Bioscience. PTX was purchased from Sigma-Aldrich. (S)-Bay K8644, nifedipine, CNQX were dissolved in DMSO and stored at −20°C before use. Other drugs were dissolved in nanopure H_2_O. All drugs were applied by perfusion in the bath solution.

## Results

### Altering LTCC activity via bilateral BLA injection of BayK at P7, P14, or P21 differentially modifies neuronal electrophysiological properties recorded at P28

**Figure 1.**
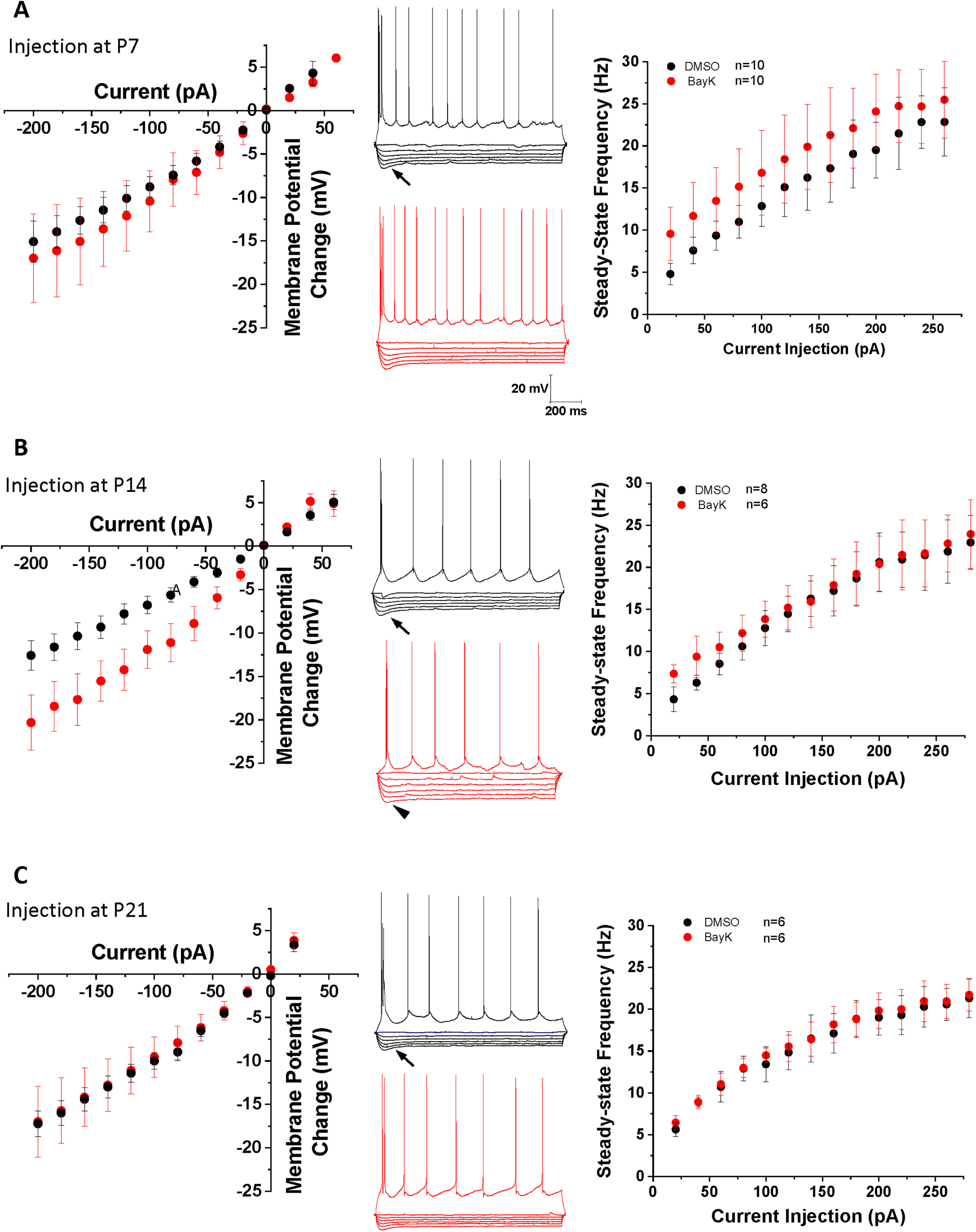
Bilateral intra-amygdala injection of BayK at early postnatal periods produces differential changes in BLA neurons intrinsic excitability at P28. *I-V* relationship plots corresponding to each experimental group are shown in the left panels (***A***, P7; ***B***, P14; ***C***, P21). Representative traces in the middle panels show voltage responses to hyperpolarizing current pulses, and a train of action potentials elicited by a suprathreshold depolarizing pulse. The arrows denote a slow depolarizing sag in the electrotonic potentials evoked by membrane hyperpolarization. A significant sag reduction (***B***, arrowhead middle traces) is observed in neurons from animals treated at P14 compared with DMSO control group. The *f-I* relationship was plotted with values of the steady-state firing frequency as a function of current injection (right panels). Animals treated with BayK at P7 showed a small increase in steady-state frequency across all current injection range, compared with the DMSO control group (***A***, right). No obvious changes were found in the *I-V* or *f-I* relationships in groups treated with BayK at P21 (***C***). Data are presented as mean ± SEM.

**Figure 2.**
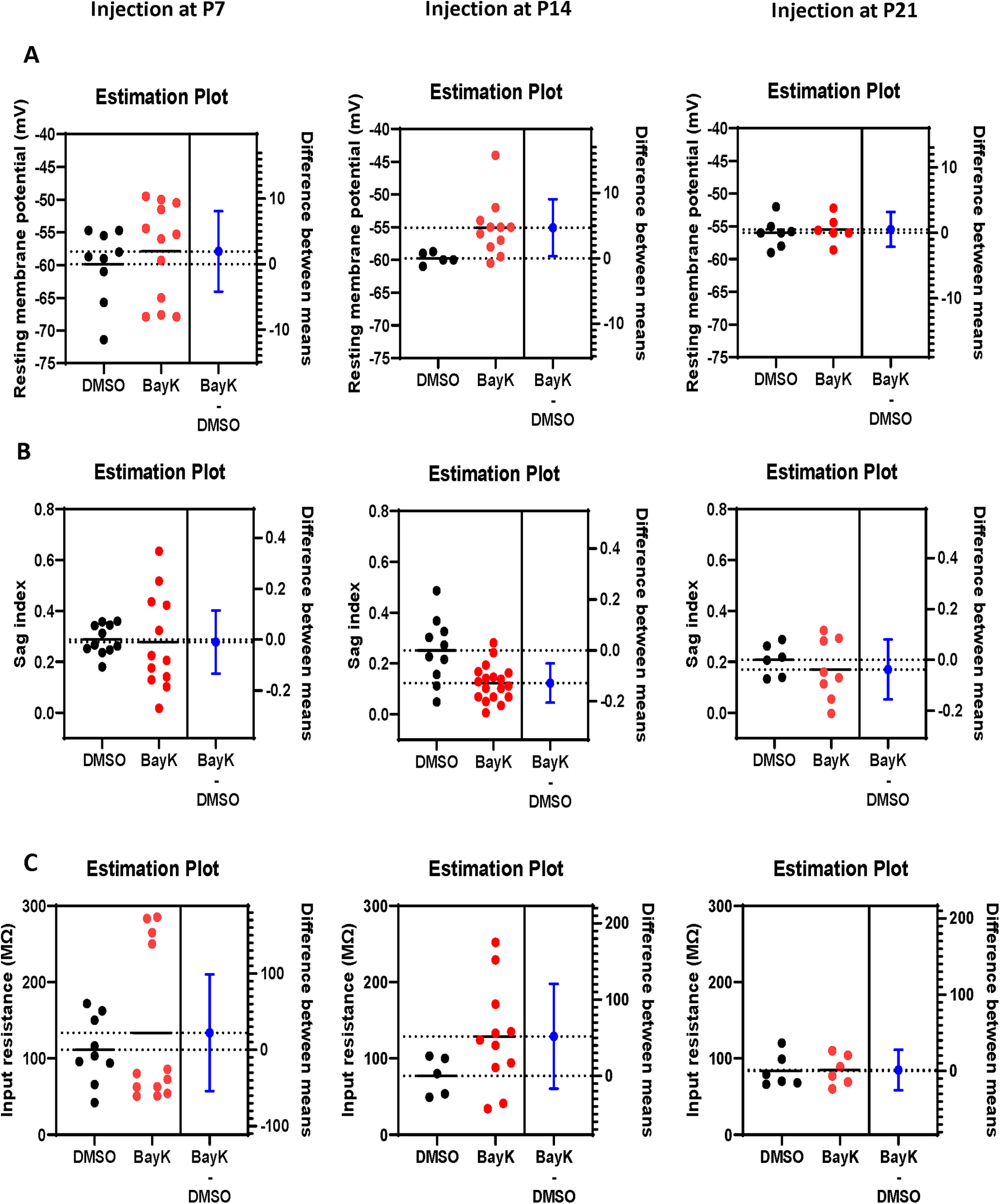
Estimation plots of BLA neuron passive membrane properties. Estimation analysis was used to compare resting membrane potential (***A***), sag index (***B***), and input resistance (***C***) in P28 rats injected with BayK (red) or DMSO (black) at P7 (left panels), P14 (middle panels), or P21 (right panels). 95% CIs are shown in blue. Resting membrane potential was increased in rats injected at P14 (mean difference between DMSO and BayK is 4.659 [95% CI 0.3014, 9.017], two tailed unpaired *t* test *t* = 2.293, df = 14, *p* = 0.0378) and the sag index was reduced (mean difference between DMSO and BayK is −0.1285 [95% CI −0.2054, −0.05154], two tailed unpaired *t* test *t* = 3.432, df = 26, *p* = 0.0020). No other properties were impacted by BayK injection.

Our previous study using brain slices showed acute BayK application to profoundly yet differentially change the intrinsic excitability of BLA neurons at distinct early postnatal ages (Zhang et al., 2020). Here, we examined whether BayK injection at P7, P14, or P21 resulted in subsequent changes to basal electrophysiological properties of BLA principal neurons at P28 (Fig. 1). In animals that received BayK injection at P14 the resting membrane potential was significantly depolarized at P28 compared with vehicle control (DMSO: −59.7 ± 0.4 mV, *n* = 5, BayK: −55.1 ± 1.3 mV, *n* = 11, *p* = 0.038, mean difference 4.659 [95% CI 0.301–9.017]; Fig. 2*A*), making it closer to the action potential threshold. In contrast, animals that received BayK treatment at either P7 or P21 did not show significant differences in resting potential at P28 compared with the DMSO group (P7, DMSO: −59.9 ± 1.9 mV, *n* = 9, BayK: −57.9 ± 2.1 mV, *n* = 12, *p* = 0.516, mean difference 1.947 [95% CI −4.205–8.099]; P21, DMSO: −55.9 ± 0.8 mV, *n* = 7, BayK: −55.5 ± 0.9 mV, *n* = 6, *p* = 0.685, mean difference 0.505 [95% CI −2.164–3.173]; Fig. 2*A*). As determined by *I-V* relations measured at P28, also notable with BayK bilateral BLA injections at P14, but not P7 or P21, was a reduction in the slow depolarizing sag observed during membrane hyperpolarizations (Fig. 1*B*, arrowhead) and a significant increase in steady-state voltage responses toward hyperpolarizing currents (Fig. 1*B*). Thus, we compared the magnitude of the sag index (Jarsky et al., 2008) and rectification ratio (Farries et al., 2005) from the *I-V* recordings at P14. The sag index was calculated as the difference between the initial maximum and the steady-state voltage deflection in response to the largest hyperpolarizing current step from the *I-V* curve, divided by the magnitude of the steady-state at the end of the current step. Neurons from DMSO treatment showed higher sag index (0.251 ± 0.041, *n* = 10) than those from BayK treatment (0.123 ± 0.016, *n* = 18; *p* = 0.002; mean difference −0.129 [95% CI −0.205 to −0.051]; estimation analysis shown in Fig. 2*B*).

P14 BayK-injected animals also exhibited an apparent but nonsignificant increase in input resistance at P28 (DMSO 77.1 ± 11.3 MΩ, *n* = 5, BayK 128.9 ± 20.6 MΩ, *n* = 11, *p* = 0.128 mean difference 51.79 [95% CI −16.87–120.4]; Fig. 2*C*). To quantitatively determine whether the apparent nonlinearity of the IV-curve in the BayK treated neurons was associated to an increase in inward rectification, we calculated the rectification ratio as the quotient of membrane resistance at negative membrane potentials divided by the resistance around resting membrane potential. We found no significant differences between the mean value of the rectification ratios from control (0.71 ± 0.06, *n* = 5) and BayK group (0.67 ± 0.05, *n* = 9; *p* =0.655). Input resistances were not significantly different in P7 (DMSO: 111.3 ± 14.6 MΩ, *n* = 9, BayK: 133.6 ± 29.5 MΩ, *n* = 12, *p* = 0.550, mean difference 22.26 [95% CI −54.20–98.72]) or P21 injection groups (DMSO: 83.67 ± 8.8 MΩ, *n* = 6, BayK: 84.8 ± 8.1 MΩ, *n* = 6, *p* = 0.924, mean difference 1.167 [95% CI −25.41–27.74]; Fig. 2*C*).

**Figure 3.**
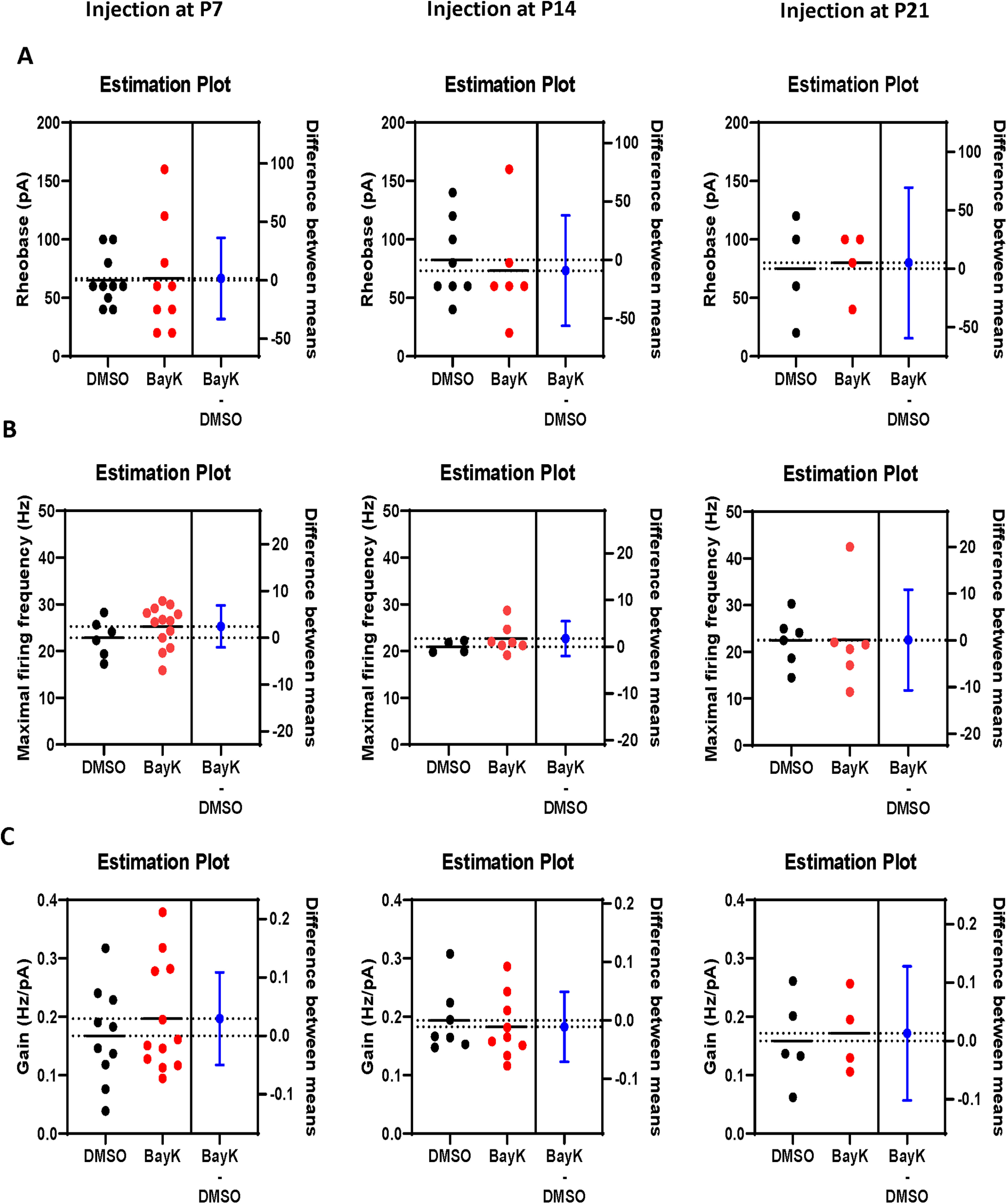
Estimation plots of BLA neuron firing properties. Estimation analysis was used to compare rheobase (***A***), maximal firing frequency (***B***), and gain (***C***) in P28 rats injected with BayK (red) or DMSO (black) at P7 (left panels), P14 (middle panels), or P21 (right panels). 95% CIs are shown in blue. No parameters were significantly different between BayK and DMSO injected groups.

To further examine the effects of postnatal BayK injection on BLA neuronal excitability, *f-I* relations (steady-state firing frequency vs current injection) were examined at P28. Results show that the rheobase was not significantly changed at P28 for BayK injection at any of the postnatal ages (P7, DMSO: 65.0 ± 6.9 pA, *n* = 10, BayK: 66.7 ± 15.63 pA, *n* = 9, *p* = 0.921 mean difference 1.667 [95% CI −33.09–36.42]; P14, DMSO: 82.5 ± 12.2 pA, *n* = 8; BayK: 73.3 ± 19.1, *n* = 6, *p* = 0.679 mean difference −9.167 [95% CI −56.34–38.00]; P21, DMSO:75.0 ± 22.2 pA, *n* = 4; BayK: 80.0 ± 14.1 pA, *n* = 4, *p* = 0.856 mean difference 5.000 [95% CI −59.35–69.35]; Fig. 3*A*). Although the p value did not reach significance level, BLA neurons from animals treated with BayK at P7 exhibited slightly higher steady-state firing frequency across the entire range of current injection tested (Fig. 1*A*, right; maximal frequency values, DMSO: 22.8 ± 1.6 Hz, *n* = 6, BayK: 25.3 ± 1.2 Hz, *n* = 13, *p* = 0.267, mean difference 2.440 [95% CI −2.050–6.929]). No changes were found in the animals treated at P14 (Fig. 1*B*, right; DMSO: 20.9 ± 0.63 Hz, *n* = 4, BayK: 22.7 ± 1.2 Hz, *n* = 7, *p* = 0.322, mean difference 1.729 [95% CI −2.003–5.461]) or P21 (Fig. 1*C*, right; DMSO: 22.5 ± 2.2 Hz, *n* = 6, BayK: 22.5 ± 4.3 Hz, *n* = 6, *p* = 0.992, mean difference 0.051 [95% CI −10.74–10.84]; Fig. 3*B*). Measuring the initial slope of the *f-I* curve indicated that the gain was not significantly different between animals treated with BayK or DMSO at P7 (DMSO: 0.17 ± 0.02 Hz/pA, *n* = 10; BayK: 0.19 ± 0.03; *n* = 12, *p* = 0.450 mean difference 0.0293 [95% CI −0.050–0.109]), P14 (DMSO: 0.19 ± 0.02 Hz/pA, *n* = 7; BayK: 0.18 ± 0.02 Hz/pA, *n* = 9, *p* = 0.697 mean difference −0.011 [95% CI −0.071–0.049]), or P21 (DMSO: 0.16 ± 0.03, *n* = 5, BayK: 0.17 ± 0.04; *n* = 4, *p* = 0.798 mean difference 0.013 [95% CI −0.102–0.128]; Fig. 3*C*), indicating that overall sensitivity in response to electrical stimuli is not affected by BayK injection. Overall, these results point to qualitative differences between the effects of acute L-type-mediated regulation of BLA intrinsic excitability and membrane properties (Zhang et al., 2020), and the effects of a single *in vivo* injection of an L-type channel agonist which raises the possibility that compensatory mechanisms operating during the timeframe between BayK treatment and electrophysiological recordings could oppose sustained hyperexcitability. Although the exact mechanism remains to be determined, this observation is consistent with a previous study showing that conditional CNS deletion of Ca_v_1.2 channel was less effective than acute intraventricular administration of isradipine in altering the acquisition of conditioned fear and LTP induction in thalamocortical inputs to the LA (Langwieser et al., 2010).

### Bilateral BLA BayK injection at early postnatal ages produces long-lasting alterations to juvenile behavioral patterns

**Figure 4.**
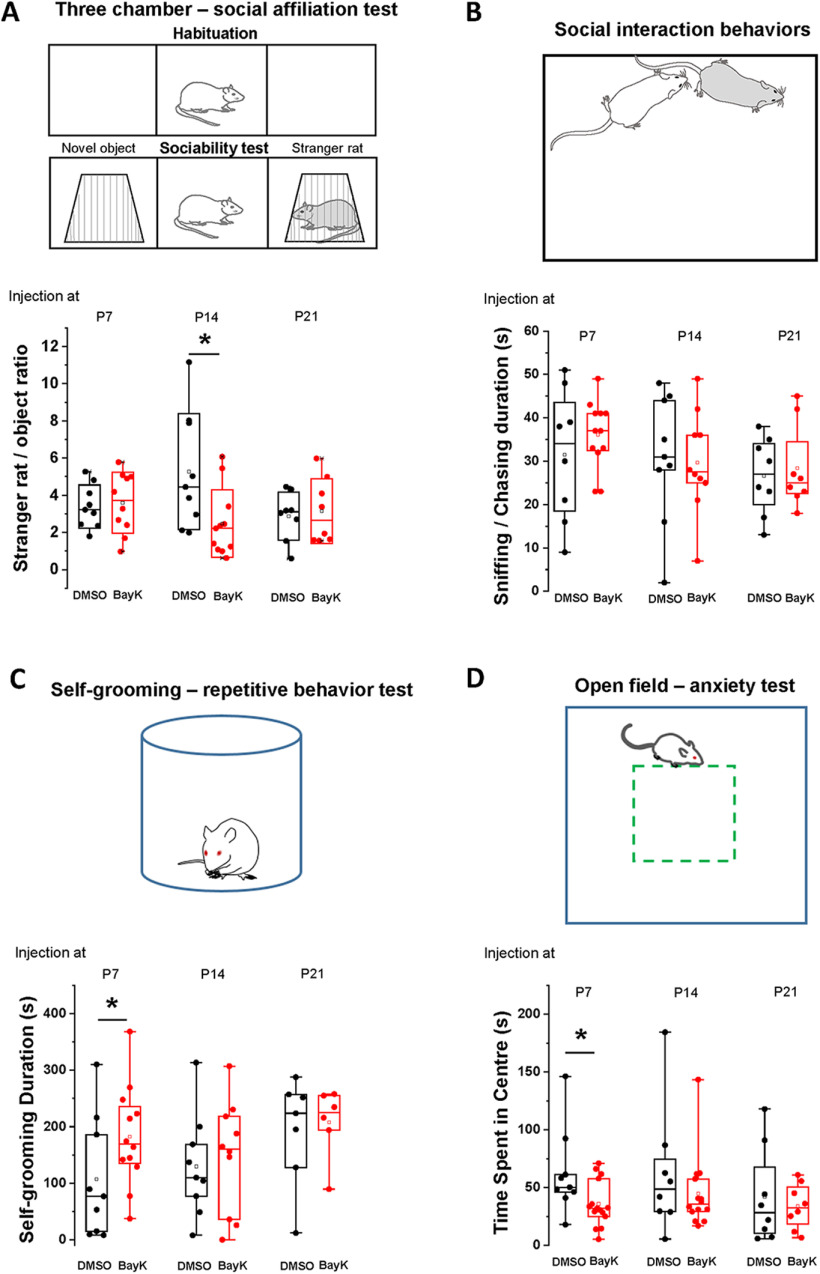
BayK injection at early developmental stages produced long-term alterations in amygdala-related behavioral phenotypes. Four different behavioral tests were performed in P28 juvenile rats previously treated at different ages with BayK or DMSO as vehicle control. Simplified schematic diagrams in the upper part of each panel indicate the experimental paradigms, where the white rat indicates the experimental subject and the dark rat indicates the naive animal used as stranger: ***A***, three-chamber social affiliation test; ***B***, social interaction behavior; ***C***, repetitive behavior; ***D***, anxiety test. Corresponding parameters are shown in black symbols for control (DMSO) and red symbols for experimental (BayK) groups. ***A***, The social affiliation was measured by the ratio of time the experimental rat spent around the stranger, over the time spent around the novel object (empty cage used as nonsocial object) and values are shown in box plots. Animals that received BayK injection at P7 (DMSO *n* = 9, BayK *n* = 10) or P21 (DMSO *n* = 8, BayK *n* = 8) showed no significant differences in this parameter compared with control, whereas BayK treatment at P14 have significant lower stranger/object ratio (DMSO *n* = 9, BayK *n* = 11), indicating a reduced social affiliation. ***B***, Social interactions were monitored for 10 min and the duration of periods of chasing/sniffing exploratory behaviors was recorded. Box plots show that BayK treatment did not induce significant differences in any age group on this behavioral parameter. ***C***, Repetitive behavior was measured as the duration of self-grooming in a restricted space. Box plots show that animals receiving BayK injection at P7 exhibited an increase in the duration of self-grooming when tested at P28 (***C***, left bars), whereas no differences were found in the P14 and P21 experimental groups. ***D***, Levels of anxiety were evaluated using an open field test and measured as the time spent in the central area. Animals with BayK treatment at P7 spent less time in the center, indicating an increased anxiety level compared with the DMSO group. No differences were found in the P14 and P21 experimental groups. Data are presented as mean ± SEM; *p < 0.05.

It was of interest and relevance to determine whether postnatal BayK injection into the BLA affected subsequent behaviors in young adult rats. Several behavioral tests are commonly used for animal models of ASD, including the three-chamber test, social interaction and self-grooming test. The three-chamber paradigm, also known as Crawley’s sociability and preference for social novelty, has been successfully employed to study social affiliation in rodents (Kaidanovich-Beilin et al., 2011). We first measured the ratio of the total time a subject rat spent in the chamber of a stranger rat over the time in chamber of the novel object. At P28, animals that had been treated with BayK at P14 showed a decreased ratio compared with DMSO treatment (Fig. 4*A*; DMSO: 5.27 ± 0.94, *n* = 9; BayK: 2.48 ± 0.55, *n* = 11; *p* = 0.022), whereas no significant differences were observed between BayK and DMSO treatment for either the P7 group (DMSO: 3.49 ± 0.36, *n* = 9; BayK: 3.59 ± 0.52, *n* = 10, *p* = 0.76) or the P21group (DMSO: 3.37 ± 1.12, *n* = 8; BayK: 3.71 ± 0.61, *n* = 8, *p* = 0.73). Measuring the time a subject rat spent in the chamber of stranger rat, there was found to be a significant decrease induced by BayK treatment at P14 compared with DMSO treatment (DMSO: 425.85 ± 20.7, *n* = 9, BayK: 338.27 ± 27.7, *n* = 11; *p* = 0.047), no significant difference between BayK and DMSO treatment in the experimental group of P7 (DMSO: 400.2 ± 19.1, *n* = 9, BayK: 356.33 ± 44.4, *n* = 10; *p* = 0.19) or P21(DMSO: 374 ± 37.4, *n* = 8, BayK: 393.46 ± 36.9, *n* = 8; *p* = 0.72). Similarly, for animals that received BayK treatment at P14, a corresponding increase was observed in the time spent in the chamber of the novel object (DMSO: 102.9 ± 15.3, *n* = 9, BayK: 178.2 ± 27.4, *n* = 11; *p* = 0.037). Again, no significant differences were found for the P7 (DMSO: 126.3 ± 9.9, *n* = 9, BayK: 131.8 ± 15.2, *n* = 10; *p* = 0.76) and P21 groups (DMSO: 152 ± 31.7, *n* = 8, BayK: 133.7 ± 24.5, *n* = 8; *p* = 0.66). The results indicate that at P14, but not P7 or P21, increased BLA LTCC activity mediated via BayK injection results in impaired social motivation toward stranger rats and an increased interest in novel objects at P28. The altered behaviors at P14 are those suggested to resemble certain phenotypes associated with ASD.

We next examined whether BayK injection during early development affected P28 rats with regard to social interaction. The total duration a subject rat spent on chasing/sniffing a stranger rat, a typical pattern of social exploration, was examined although no significant differences were found between BayK and DMSO treatments in any of the experimental groups (Fig. 4*B*; P7 DMSO: 31.5 ± 5.36 s, *n* = 8, BayK: 36.1 ± 2.2 s, *n* = 12, *p* = 0.38; P14 DMSO: 30.9 ± 4.9 s, *n* = 9, BayK: 29.7 ± 3.7 s, *n* = 10, *p* = 0.84; P21 DMSO: 26.6 ± 3.1, *n* = 8, BayK: 28.4 ± 3.4, *n* = 8, *p* = 0.71). Also measured was the total duration of climbing up on the back of a stranger rat as an index of active social interaction (data not shown), no significant differences were found in the three experimental groups.

The occurrence of repetitive behavior was also examined (Fig. 4*C*). Animals treated with BayK at P7 showed a significant increase in self-grooming duration at P28 compared with DMSO treated animals (DMSO: 107.1 ± 35.6 ms, *n* = 9, BayK: 182.5 ± 25.7 ms, *n* = 11, *p* = 0.038), indicating a higher level of repetitive behavior. Contrastingly, no differences were found for the P14 or P21 experimental groups (P14, DMSO: 129.6 ± 30.1 ms, *n* = 9, BayK: 147.2 ± 31.3 ms, *n* = 10, *p* = 0.69; P21, DMSO: 193.9 ± 36.3 ms, *n* = 7, BayK: 207.1 ± 25.3 ms, *n* = 6, *p* = 0.76).

Human and animal studies indicate important roles for the amygdala in mediating fear and anxiety, and in the manifestation of anxiety disorders (Forster et al., 2012), thus we also examined the effects of BLA BayK injection on long-term anxiety in juvenile rats (Fig. 4*D*). The results show that animals treated with BayK at P7 (DMSO: 62.5 ± 12.3 ms, *n* = 9, BayK: 35.9 ± 5.1 ms, *n* = 15; *p* = 0.03) exhibit a significant decrease in time spent in the center of the area, indicating a higher level of anxiety at P28 compared with DMSO treatment. No significant differences were found for animals that received treatments at either P14 (DMSO: 61.9 ± 19.5 ms, *n* = 8, BayK: 44.8 ± 8.5 ms, *n* = 14; *p* = 0.36) or P21 (DMSO: 42.1 ± 14.6 ms, *n* = 8, BayK: 33.9 ± 6.9 ms, *n* = 8, *p* = 0.62).

Taken together, behavioral studies at P28 following bilateral injection of BayK to rat pups showed distinct long-term behavioral alterations; BayK at P7 produced higher anxiety levels and repetitive behaviors, while BayK at P14 induced an impaired ability for social affiliation, together suggesting the crucial but differential involvement of LTCC activity in BLA circuits underlying the development of behavioral phenotypes. Interestingly, BayK injection into the BLA at P21 had no significant behavioral effects at P28 on any of the behaviors examined. The changes observed for the P7 and P14 experimental groups suggest the existence of a vulnerable period during early development, while the lack of observable effects at P21 may reflect that neural circuits underlying these behaviors have already reached a stage where they are less sensitive to changes in LTCC activity.

### Altering LTCC activity at distinct postnatal stages differentially affects LTP in BLA neurons

The LTP of synaptic activity is the most widely studied form of synaptic plasticity and plays important roles in learning, memory formation, fear, stress and social activities (Jung et al., 2013; Leuner and Shors, 2013; Nicoll, 2017). Here, we examined whether BayK injection into the BLA at P7, P14, and P21 mediated changes in synaptic plasticity at P28 regarding excitatory inputs from the LA into the BLA.

**Figure 5.**
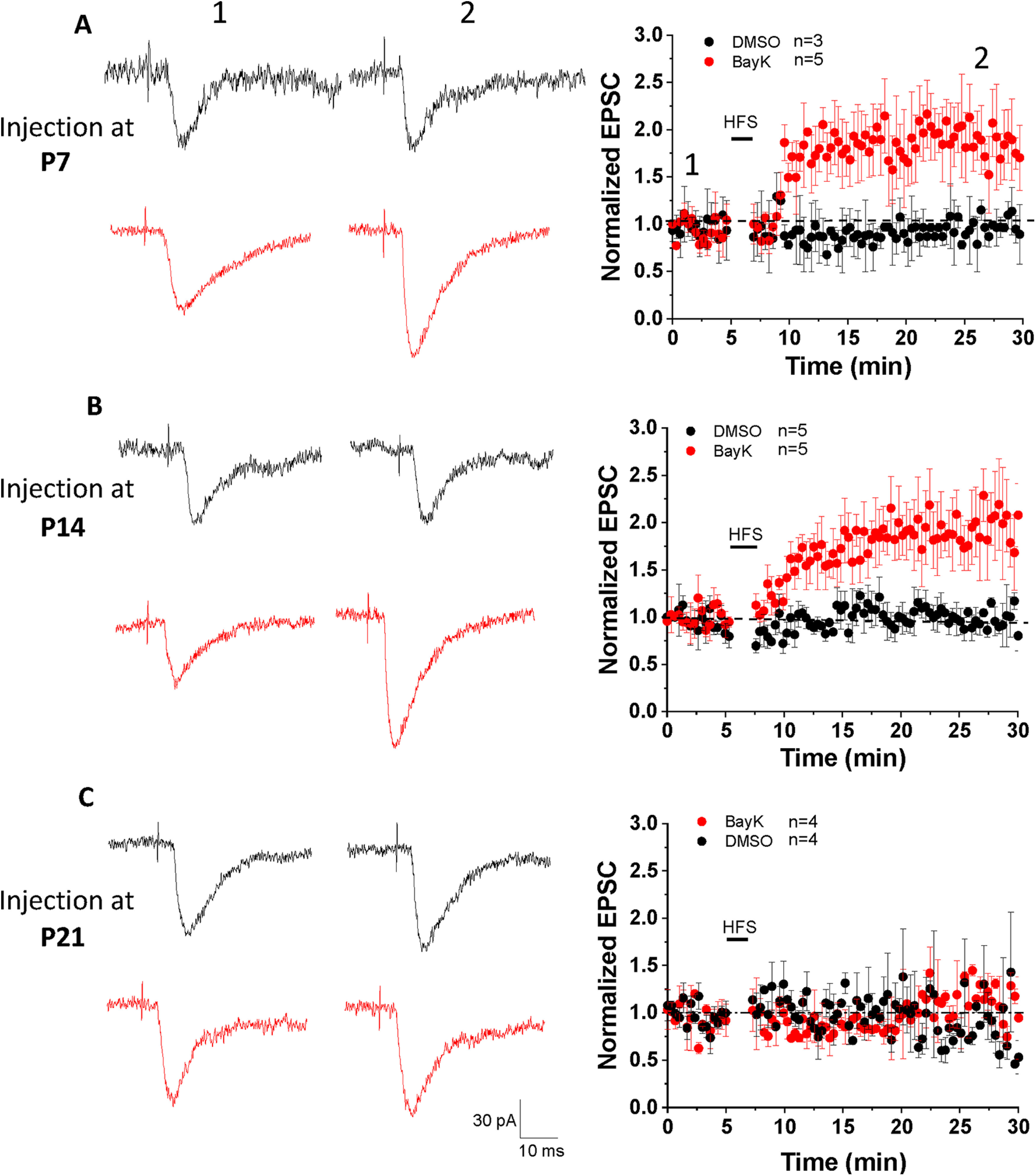
LTP was induced in P28 BLA neurons from rats that received bilateral BayK injection at P7 and P14, but not at P21. EPSC responses in BLA neurons were elicited by stimulating LA at 0.05 Hz. A HFS at 100 Hz (1 s duration, repeat 5 times at 0.1 Hz, membrane potential was holding at +30 mV) was used to induce LTP. The representative traces in the left panels show single EPSCs recorded before (left) and 25 min after (right) HFS. Black traces represent recordings in control groups while red traces represent BayK groups. EPSC amplitudes are presented as normalized values relative to the baseline amplitude before HFS. LTP was induced in the animals that received BayK treatment at P7 (***A***) or P14 (***B***) compared with their DMSO counterparts. No LTP was observed from animals treated with BayK at P21 (***C***). Data are presented as mean ± SEM.

**Figure 6.**
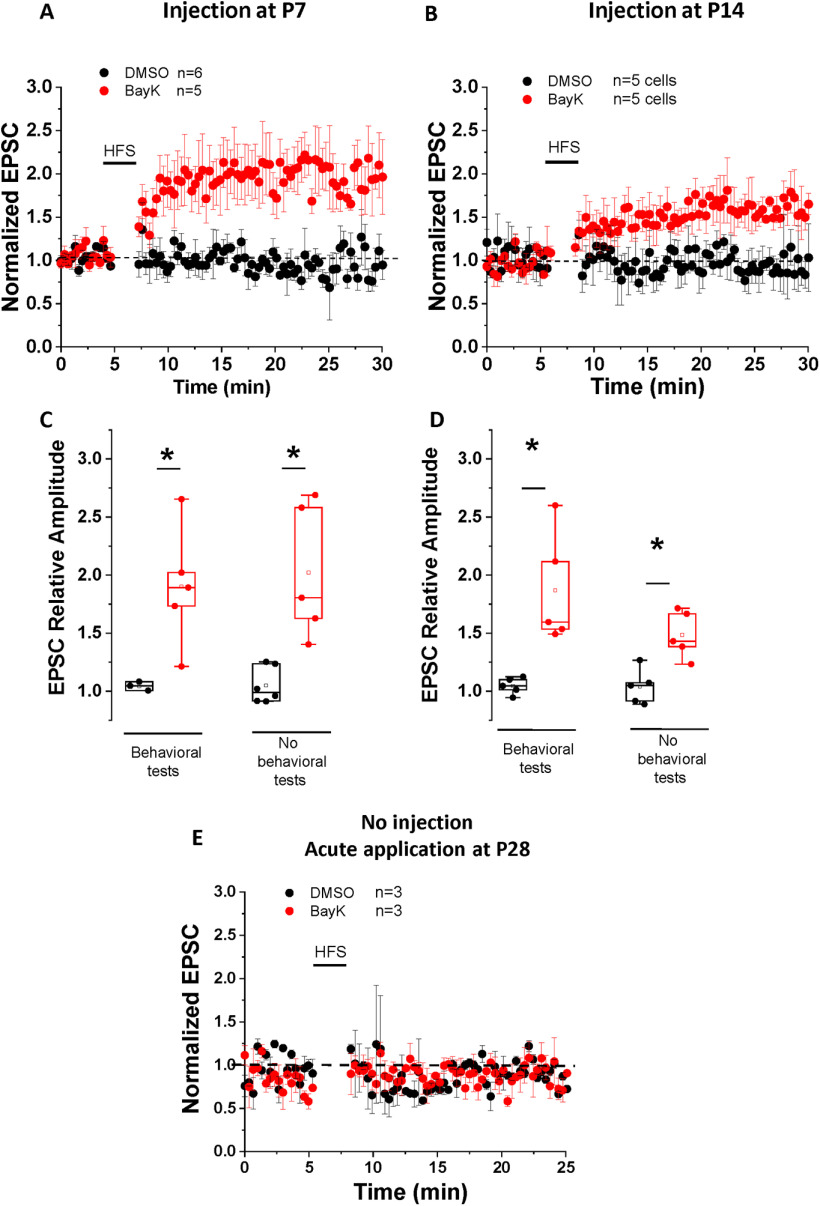
Changes in synaptic strength induced after BayK treatment at P7 or P14 occur without preceding behavioral tests. Panels ***A***, ***B*** show data from P28 animals treated with BayK or DMSO at P7 or P14 with no previous behavioral tests performed. EPSC amplitudes are presented as normalized values relative to the baseline amplitude before HFS. HFS paired with postsynaptic depolarization induced LTP that was significantly different between BayK and DMSO control in both P7 (***A***) and P14 (***B***) experimental groups. Box plots in panels ***C***, ***D*** show the relative EPSC amplitude at 25 min after the HFS, indicating that LTP was successfully induced in neurons from animals that were treated with BayK at P7 or P14, regardless of previous performance of behavioral tests. ***E***, Ratio of EPSC amplitude relative to baseline before HFS obtained from naive noninjected P28 male rats. Acute BLA slices were perfused in ACSF containing 2 μm BayK for 10 min before baseline stimulation. Under these experimental conditions BayK did not induce LTP. Data are presented as mean ± SEM; *p < 0.05.

In the presence of GABA receptor blockers PTX and CGP52432, single-pulse stimulation at low frequency applied in the LA elicited EPSC responses in BLA neurons (recorded as basal synaptic activity during a 5-min period; Fig. 5, left representative traces). To induce LTP we applied HFS (1-s train of repetitive pulses at 100 Hz, repeated 5 times at 0.1 Hz) paired with membrane potential depolarization to +30 mV. Under these conditions LTP was not induced in neurons from vehicle-treated animals at any of the P7, P14, or P21 ages. In contrast, HFS induced an increase in EPSC amplitude in BLA neurons from animals treated with BayK at both P7 and P14 (Fig. 5*A*,*B*) but not P21 (Fig. 5*C*). LTP in BLA neurons following BayK treatment at P7 showed a significant increase in EPSC amplitude over the 30 min recording period (Fig. 5*A*; normalized EPSC amplitude at 25 min: DMSO 0.97 ± 0.08, *n* = 3, BayK 1.84 ± 0.14, *n* = 5, *p* = 0.005; EPSC amplitude at baseline: 126.18 ± 11.9 pA for DMSO and 131.5 ± 17.1 pA for BayK, EPSC amplitude at 25 min of recording: 122.9 ± 19.6 pA for DMSO, and 241.7 ± 36 pA for BayK). Similarly, LTP in neurons from animals treated with BayK at P14 was significantly enhanced (Fig. 5*B*; normalized EPSC amplitude at 25 min: DMSO 0.99 ± 0.09, *n* = 5, BayK 1.86 ± 0.09, *n* = 5, *p* = 0.004; EPSC amplitude at the baseline: 137.9 ± 19.3 pA for DMSO and 123.3 ± 21.3 pA for BayK, EPSC amplitude at 25 min of recording: 136.9 ± 19.7 pA for DMSO, and 226.3 ± 30.1 pA for BayK). LTP could not be induced in neurons from either the P21 vehicle or BayK groups (Fig. 5*C*; normalized EPSC amplitude at 25 min: DMSO 0.96 ± 0.12, *n* = 4, BayK 1.02 ± 0.11, *n* = 4, *p* = 0.71; EPSC amplitude at baseline: 102 ± 17.5 pA for DMSO and 121.6 ± 20.1 pA for BayK, EPSC amplitude at 25 min of recording: 98.8 ± 26 pA for DMSO and 124.2 ± 12.7 pA for BayK). Importantly, BayK application on BLA slices from naive animals did not result in the induction of LTP using the HFS protocol (Fig. 6*E*), suggesting that activation of acute LTCC activation alone is not enough for LTP induction.

### LTCC-associated LTP induction is enhanced but not determined by prior behavioral experience

Environmental novelty and/or prior exposure to behavioral testing can impact the subsequent induction of LTP (Roman et al., 1999; Moncada and Viola, 2007; Eckert and Abraham, 2010). In a valproate rat model of ASD the induction of LTP for thalamic inputs into the LA occurred following behavioral experiments measuring fear conditioning and extinction with an identical LTP observed in the absence of preceding behavioral tests (Lin et al., 2013). Here, we asked whether the LTP observed in BayK-treated animals could have resulted as a consequence of the behavioral experience itself during assessment of behavioral traits, and also whether BayK treatment *per se* could cause permissive functional changes for LTP to occur in the intra-amygdala circuit.

First, we performed slice patch-clamp recordings on P28 rats that received BayK or vehicle injections at P7 without any behavioral tests or environmental disturbances before the recordings. No significant differences in voltage-current relationship, input resistance and resting membrane potentials between BayK and DMSO experimental groups were detected (data not shown). LTP induction experiments (Fig. 6*A*) showed similar results when compared to recordings performed after behavioral tests (Fig. 6*C*; EPSC relative amplitude at 25 min: DMSO 1.05 ± 0.07, *n* = 6, BayK 2.02 ± 0.26, *n* = 5, *p* = 0.003). These data indicate that BayK treatment alone is sufficient to produce functional changes in the observed electrophysiological properties.

In P28 animals that received BayK or DMSO injections at P14 without behavioral tests or environmental disturbance before the recordings, similar quantitative differences compared with the experimental groups with behavioral tests were observed: BLA neurons from animals treated with BayK displayed reduced inward rectification in the *I-V* relation and an upward shift in the *f-I* relationship but with no change in input resistance (data not shown). The data indicate that without performing behavioral tests the effects of BayK on LTP induction (Fig. 6*B*,*D*; normalized EPSC amplitude at 25 min: DMSO 0.85 ± 0.1, *n* = 4, BayK 1.54 ± 0.18, *n* = 4, *p* = 0.019) were more moderate compared with results following behavioral testing (Fig. 6*D*), consistent with previous findings that behavioral experiences can modify synaptic plasticity. Overall, the data support the notion that LTCC-driven activity changes within BLA circuitry are sufficient to induce LTP albeit prior behavioral experience can further modify the magnitude of the LTCC-mediated effects. It is important to note that the acute application of BayK *in vitro* was not sufficient to elicit an increase in synaptic strength at the LA to BLA input in response to HFS paired with postsynaptic depolarization (Fig. 6*E*).

### Increasing LTCC activity during development alters the expression of ionotropic glutamatergic receptor genes

**Figure 7.**
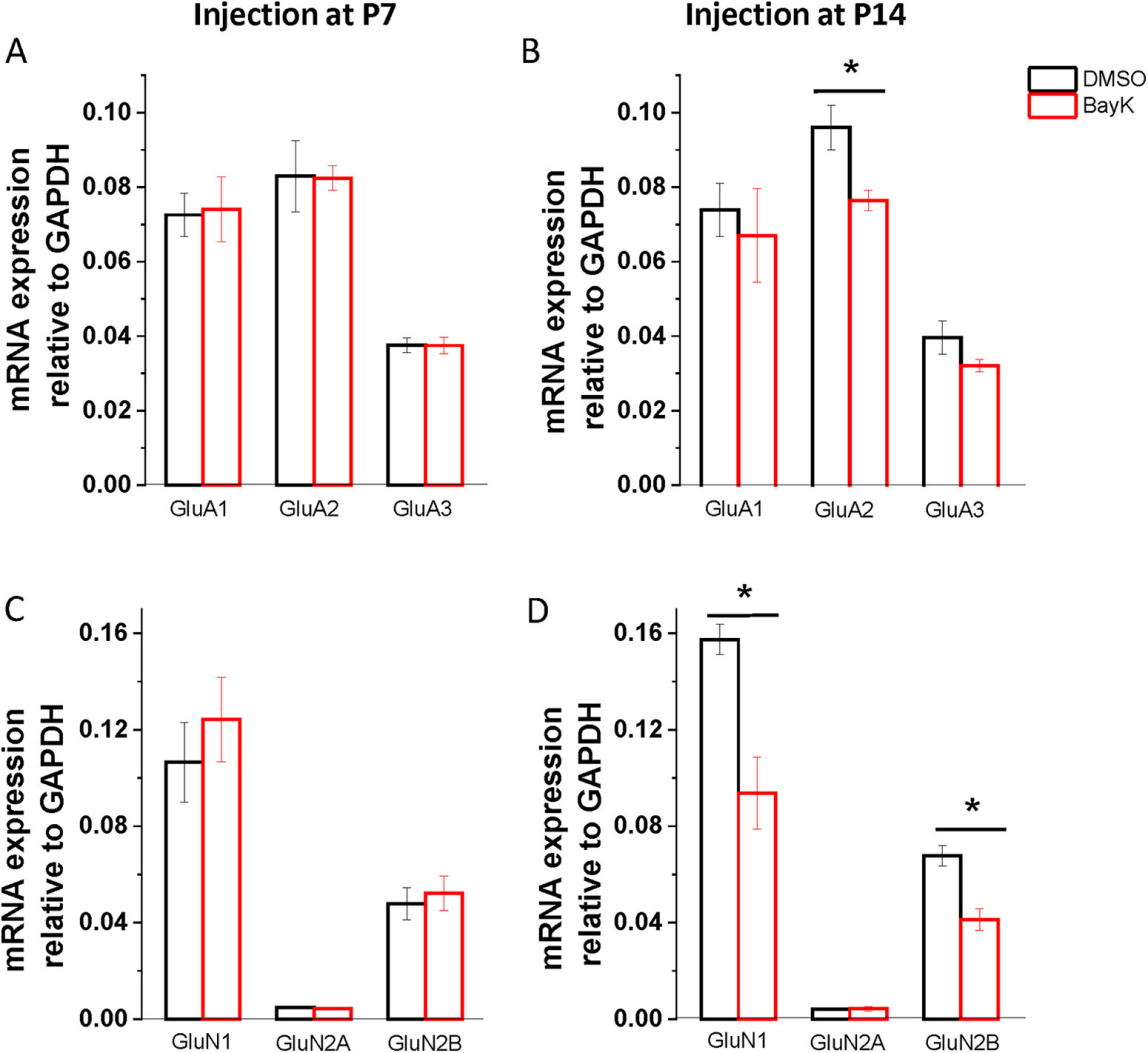
Bilateral microinjection of BayK during development has long-term consequences on mRNA expression of NMDA and AMPA receptor subunits. BLA samples were collected at P28 from rats injected with BayK (red) or DMSO (black) at P7 (left) or P14 (right), and mRNA expression of glutamatergic receptors AMPA (upper panel) and NMDA (lower panel) was quantified by RT-PCR. No significant changes were observed in rats injected at P7 for the expression of both AMPA and NMDA receptor subunits (***A***, ***C***). In rats injected with BayK at P14, expression of AMPA receptor subunit GluA2 was significantly decreased (***B***; *p* = 0.032). Further, NMDA receptor subunits GluN1 and GluN2B expression was also decreased (***D***; GluN1 *p* = 0.019; GluN2B *p* = 0.011). mRNA expression was calculated as a ratio to GAPDH and expressed as mean ± SEM. Asterisks indicate significant difference between BayK and control group using ANOVA and *post hoc* Bonferroni test.

The subunit composition of ionotropic glutamatergic receptors can influence synaptic plasticity. For instance, the AMPA receptor subunit GluA2 has been implicated in the induction of LTP (Jia et al., 1996; Whitehead et al., 2013). While for NMDA receptors, the ratio of GluN2A/2B in adults has been suggested to regulate the threshold for synaptic plasticity (Yashiro and Philpot, 2008; Paoletti et al., 2013). Given that we found an increase in the induction of LTP in rats injected at P7 and P14, we hypothesized that BayK injection at these critical periods might lead to changes in the subunit composition of ionotropic glutamatergic receptors. Here, we examined the mRNA expression of AMPA and NMDA receptor subunits at P28 in rats injected at P7, P14, and P21. There were no significant expression changes in animals treated with BayK at P7 for AMPA receptor subunits (Fig. 7*A*; GluA1, DMSO 0.073 ± 0.006, *n* = 4, BayK 0.074 ± 0.09, *n* = 4, *p* = 0.889; GluA2, DMSO 0.083 ± 0.009, *n* = 4, BayK 0.082 ± 0.003, *n* = 4, *p* = 0.961; GluA3, DMSO 0.038 ± 0.002, *n* = 4, BayK 0.037 ± 0.002, *n* = 4, *p* = 0.985) or NMDA receptor subunits (Fig. 7*C*; GluN1, DMSO 0.107 ± 0.02, *n* = 4, BayK 0.124 ± 0.017, *n* = 4, *p* = 0.488; GluN2A, DMSO 0.005 ± 0. 0004, *n* = 4, BayK 0.004 ± 0.0003, *n* = 4, *p* = 0.45; GluN2B, DMSO 0.048 ± 0.007, *n* = 4, BayK 0.052 ± 0.007, *n* = 4, *p* = 0.672). Contrastingly, in the P14 experimental groups, the expression of AMPA GluA2 subunits was lower in BayK-injected rats than control (Fig. 7*B*; DMSO 0.096 ± 0.006, *n* = 4, BayK 0.076 ± 0.003, *n* = 4, *p* = 0.032), and no differences were found in GluA1 (DMSO 0.074 ± 0.007, *n* = 4, BayK 0.067 ± 0.012, *n* = 4, *p* = 0.692) or GluA3 (DMSO 0.039 ± 0.004, *n* = 4, BayK 0.032 ± 0.002, *n* = 4, *p* = 0.17). The expression of NMDA GluN1 and GluN2B subunits were also significantly lower in rats injected with BayK at P14 (Fig. 7*D*; GluN1: DMSO 0.157 ± 0.006, *n* = 4, BayK 0.094 ± 0.015, *n* = 4, *p* = 0.019; GluN2B: DMSO 0.067 ± 0.004, *n* = 4, BayK 0.04 ± 0.004, *n* = 4, *p* = 0.012), whereas no difference was present in the expression of GluN2A (DMSO 0.004 ± 0.0004, *n* = 4, BayK 0.004 ± 0.001, *n* = 4, *p* = 0.909). As such, the enhanced ability to undergo LTP at P28 for rats injected at P14 may be linked to regulation of functional synaptic receptors expressed in glutamatergic synapses in the BLA.

### Expression of IEGs is transiently altered by BayK bilateral injection at early postnatal ages

Transient expression of IEGs can translate into long-term physiological effects and we hypothesized that a BayK-mediated increase in calcium influx may induce a transcriptional response in relevant BLA circuits. Here, we examined the expression of IEGs as indirect reporters of LTCC-mediated calcium influx evoked by intra-amygdala infusion of BayK. qRT-PCR was performed to determine mRNA levels of the rapid response IEGs *c-fos*, *arc*, and *Homer1a* in BLA samples obtained at different time intervals after intra-BLA infusion of BayK.

**Figure 8.**
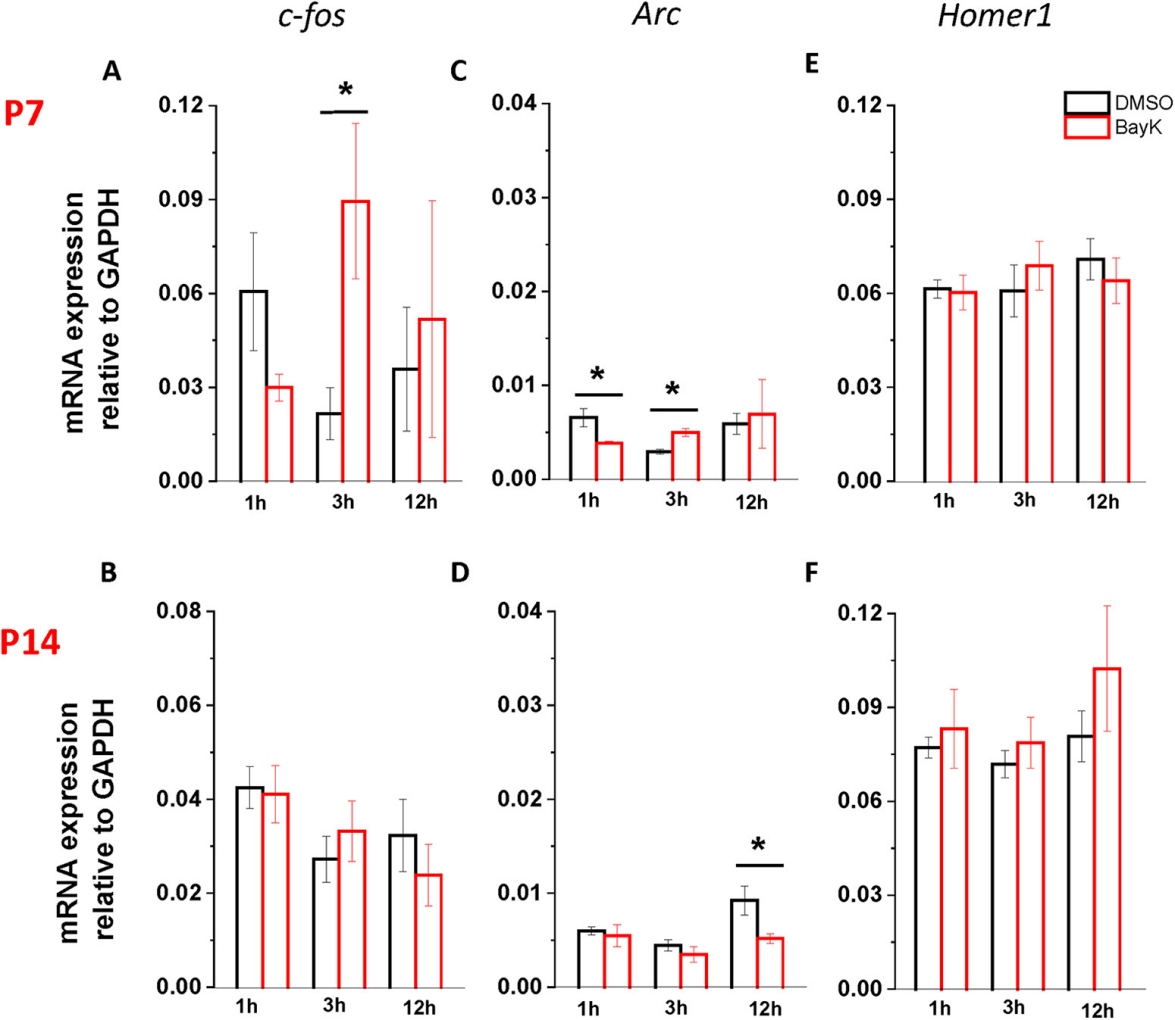
Quantification of IEGs mRNA expression in BLA at different time points after local application of BayK. Amygdala samples were collected at P7 (upper panels) or P14 (lower panels), at three different time points (1, 3, and 12 h) after local microinjection of BayK (red) or DMSO vehicle (black), and relative gene expression of *c-fos* (left), *arc* (middle), and *Homer1A* (right) was quantified by RT-PCR. Significantly increased levels of c-fos mRNA expression following intra-amygdala infusion of BayK were observed in tissue from P7-treated rat pups (***A***). After a mild drop 1 h after BayK injection, increased mRNA levels were seen at 3 h, followed by a reduction to control values at 12 h. *Arc* exhibited very low levels of expression at both ages, and showed no increase after BayK exposure (***C***, ***D***). *Homer 1a* mRNA levels remained unchanged regardless of treatment (***E***, ***F***). Individual samples were processed in triplicate, and results of each time point were obtained from four independent experiments (*n* = 12 animals per target gene). Average mRNA expression was calculated as ratios of IEGs to GAPDH and values are expressed as means ± SEM. Asterisks indicate significant difference between BayK and control group using ANOVA and *post hoc* Bonferroni test, with a level of significance of 0.041 for P7 c-fos expression at 3 h; 0.032 and 0.006 for P7 Arc at 1 and 3 h, respectively, and 0.048 for P14 Arc at 12 h.

*c-fos* IEG has been widely used as an indirect reporter of neuronal activity as its expression is triggered by calcium influx through both activated NMDA receptors and LTCCs (Rajbhandari et al., 2016; Hudson, 2018). P7 rat pups showed a slightly decreased *c-fos* mRNA expression 1 h after BayK injection (Fig. 8*A*; DMSO 0.061 ± 0.019, *n* = 4, BayK 0.030 ± 0.004, *n* = 4, *p* = 0.163), that was followed by a significant increase at 3 h after injection (Fig. 8*A*; DMSO 0.022 ± 0.008, *n* = 4, BayK 0.089 ± 0.025, *n* = 4, *p* = 0.041). The level of *c-fos* expression was back to control levels at 12 h (Fig. 8*A*; DMSO 0.036 ± 0.02, *n* = 4, BayK 0.052 ± 0.038, *n* = 4, *p* = 0.72). Notably, P14 pups showed no significant differences in *c-fos* expression at any time points following BayK injection (Fig. 8*B*; 1 h DMSO 0.06 ± 0.019, *n* = 4, BayK 0.03 ± 0.004, *n* = 4, *p* = 0.856; 3 h, DMSO 0.022 ± 0.008, *n* = 4, BayK 0.09 ± 0.025, *n* = 4, *p* = 0.49; 12 h, DMSO 0.036 ± 0.017, *n* = 4, BayK 0.052 ± 0.017, *n* = 4, *p* = 0.0436).

Activity-regulated cytoskeletal (*arc*) is involved in AMPA-type glutamate receptor trafficking which regulates long-term synaptic plasticity and memory (Korb and Finkbeiner, 2011). Here, BayK injected P7 pups exhibited a transient decrease in *arc* expression at 1 h (Fig. 8*C*; DMSO 0.007 ± 0.001, *n* = 4, BayK 0.004 ± 0.0008, *n* = 4, *p* = 0.032) followed by an increase at 3 h (DMSO 0.003 ± 0.0002, *n* = 4, BayK 0.005 ± 0.0004, *n* = 4, *p* = 0.007) and that had returned to control levels at 12 h (DMSO 0.006 ± 0.001, *n* = 4, BayK 0.007 ± 0.003, *n* = 4, *p* = 0.793). Contrastingly, BayK-injected P14 exhibited a significant decrease in *arc* expression at 12 h (Fig. 8*D*; DMSO 0.009 ± 0.002, *n* = 4, BayK 0.005 ± 0.0005, *n* = 4, *p* = 0.048) with no differences at other time points (1 h, DMSO 0.006 ± 0.0004, *n* = 4, BayK 0.005 ± 0.001, *n* = 4, *p* = 0.688; 3 h, DMSO 0.004 ± 0.0005, *n* = 4, BayK 0.004 ± 0.0008, *n* = 4, *p* = 0.38).

Homer1 proteins play a significant role in the organization and intracellular signaling at glutamatergic synapses (Shiraishi-Yamaguchi and Furuichi, 2007). Homer expression is bimodal, with constitutive (Homer1b and c) and/or transient (Homer1a and Ania3) components triggered by neuronal activity (Bottai et al., 2002). Here, measuring all *Homer1* transcripts combined, there were no detectable differences between DMSO and BayK at P7 (Fig. 8*E*; 1 h, DMSO 0.061 ± 0.003, *n* = 4, BayK 0.06 ± 0.006, *n* = 4, *p* = 0.087; 3 h, DMSO 0.061 ± 0.008, *n* = 4, BayK 0.069 ± 0.008, *n* = 4, *p* = 0.51; 12 h, DMSO 0.07 ± 0.007, *n* = 4, BayK 0.064 ± 0.007, *n* = 4, *p* = 0.507), or P14 (Fig. 8*F*; 1 h, DMSO, 0.077 ± 0.003, *n* = 4, BayK, 0.083 ± 0.013, *n* = 4, *p* = 0.668; 3 h, DMSO 0.072 ± 0.004, *n* = 4, BayK 0.079 ± 0.008, *n* = 4, *p* = 0.484; 12 h, DMSO 0.081 ± 0.008, *n* = 4, BayK 0.102 ± 0.02, *n* = 4, *p* = 0.356).

## Discussion

Intrinsic excitability plays a fundamental role in neuronal information processing with firing properties being tightly regulated to maintain a functional range that allows appropriate responses to specific stimuli while also adapting to temporary fluctuations and changes during development (Schulz, 2006; Tien and Kerschensteiner, 2018). The anomalous modification of neuronal excitability has been implicated in various pathologic conditions (Beck and Yaari, 2008). For example, in a mouse model of Rett syndrome, a neurodevelopmental disorder with autistic features, changes in membrane conductance leading to hyperexcitability have been reported in locus coeruleus neurons (Taneja et al., 2009), suggesting that alteration of intrinsic properties might contribute to early onset circuit disruption.

In the rat BLA, application of BayK to acute brain slices from animals in the P7–P21 critical period of development resulted in age-dependent changes in firing properties, consistent with a crucial role of LTCCs in regulating neuronal excitability (Zhang et al., 2020). However, that application of BayK *in vivo* could trigger permanent alterations in membrane properties of BLA principal neurons which could functionally impact amygdala excitability or associated behaviors at subsequent life stages remained to be explored. In the present study, the electrophysiological properties of BLA principal neurons from juvenile rats at P28 were compared between three experimental groups; each animal cohort receiving BayK or DMSO treatment by stereotaxic bilateral microinjection at P7, P14, or P21.

At P28, there was a detectable trend toward higher firing frequency in response to current injections in principal neurons from animals injected with BayK at P7, although *f-I* parameters did not show significant statistical differences between BayK and control treatment in any of the P7, P14, or P21 experimental groups. For injections at P7 and P21, there were also no significant long-term changes in gain or rheobase at P28, thus long-term membrane excitability appears not to be affected by BayK administration at these stages.

In contrast, BayK-mediated effects were observed in P28 BLA neurons from animals treated at P14, including a more depolarized membrane resting potential compared with controls. An apparent but nonsignificant increase in Ro, in addition to a marked reduction of time-dependent sag in response to membrane hyperpolarizations was also evident at P28 in BLA neurons from P14 BayK-injected animals.

BLA circuits that mediate behaviors have been characterized using standardized paradigms that evaluate anxiety, social skills and repetitive behaviors. Here, animals receiving bilateral BLA injection at P14 exhibited a significant decrease in time spent with a stranger rat and also increased time with a novel object, indicating impairment in social affiliation abilities. In contrast, animals receiving BayK treatment at P7 or P21 did not show any significant differences compared with DMSO control groups. These results are consistent with the ontogeny of social behaviors in rats showing the emergence of social interest activities between P14 and P28 (Wood et al., 2003).

The three-chamber test provides only partial information regarding social approach since the experimental animal and the stranger rat are not allowed to freely engage in reciprocal social interaction. Therefore, the tendency to explore a conspecific and the ability to sustain social interactions was further evaluated in an open field chamber. In rodents, reciprocal face-to-face sniffing can be related to social dominance, whereas chasing and sniffing of the flank or the anogenital region are exploratory activities indicating propensity to social interaction (Wesson, 2013). Regardless of the age group tested, no significant differences were observed in the duration of sniffing/chasing behaviors of BayK-treated or DMSO-treated animals when allowed to interact with a previously unknown conspecific animal.

The self-grooming behavior of rats confined in a restricted space was also evaluated. Only animals receiving bilateral BLA injection of BayK at P7 exhibited an increased duration of self-grooming periods compared with the DMSO group. No significant differences were found in the other age groups, suggesting that the consolidation of BLA circuitry mediating repetitive grooming behaviors occurred within the first two postnatal weeks. A similar age-dependent profile was observed with the open field test, a behavioral assay measuring anxiety that has been extensively used to examine amygdala function (Ishikawa et al., 2015). Taken together, the results from the behavioral tests support the hypothesis that a functional increase in LTCC activity at certain postnatal critical periods during early neurodevelopment can induce long-term changes in behaviors.

BayK treatment might affect multiple functional levels within the amygdala circuitry. Previous data show that during the critical period of BLA development there is a temporal association in the expression of synaptic plasticity and an ability to learn behavioral responses induced by fear conditioning (Thompson et al., 2008). LTP was examined as a potential cellular mechanism underlying the behavioral alterations observed.

It is well described that local amygdala networks mediate the acquisition, expression and extinction of conditioned fear, and regulate social behaviors (Duvarci and Pare, 2014; Ferrara et al., 2021). Also, that experience-dependent increases in synaptic strength of excitatory projections from the lateral nucleus of the amygdala into the BLA (LA to BLA) controls encoding of anxiety-like behaviors and other emotion-related behavioral responses (Nonaka et al., 2014; Bocchio et al., 2017).

Here, P21 animals regardless of treatment did not exhibit LTP after HFS paired with postsynaptic depolarization. Contrastingly, BayK treatment at either P7 or P14 showed LTP induction with a two-fold amplitude increase in EPSCs in brain slices from P28 animals previously examined in behavioral tests (Figs. 5, 6).

It is known that environmental novelty and/or the exposure to behavioral tests may impact the subsequent induction of LTP (Roman et al., 1999; Moncada and Viola, 2007; Eckert and Abraham, 2010). This process, known as behavioral tagging, is thought to involve the synthesis of synaptic plasticity-related proteins important for the induction and expression of LTP (Moncada and Viola, 2007; Moncada et al., 2011). Here, we tested two additional groups of animals treated at P7 or P14 but that were not exposed to environmental novelty or behavioral testing before electrophysiological recordings. Compared with DMSO controls LTP was significantly induced in the BLA from both P7 and P14 animals that received BayK treatment (Fig. 6). Interestingly, while the P7 BayK group showed potentiation of almost identical magnitude regardless of prior exposure to behavioral experience, the magnitude of LTP in the P14 group was slightly less compared with the P14 BayK group that underwent behavioral tests (Fig. 6*B*,*D*). Overall, our results are consistent with a previous study using a valproate rat model of autism where the induction of LTP at thalamic inputs into the LA after behavioral testing, measuring fear conditioning and extinction, did not show behavioral-dependent changes in LTP (Lin et al., 2013).

The data suggest that pharmacological activation of LTCCs during the P7 to P14 critical period produces long-term changes in synaptic plasticity which cannot be replicated in an adult BLA synapse by direct application of an LTCC agonist. It is also possible that the permissive functional changes driving LTP induction at P14 can be further influenced by behavioral tagging triggered by behavioral experiences.

The proportion and subunit composition of ionotropic glutamatergic receptors appears plastic and can undergo changes during development (Lopez de Armentia and Sah, 2003) and with life experiences (Suvrathan et al., 2014; Yi et al., 2017). In the amygdala, stress can generate NMDA containing silent synapses (Suvrathan et al., 2014) as well as alter the expression of AMPA receptor subunits (Yi et al., 2017; Guadagno et al., 2020), resulting in enhanced synaptic plasticity. Given the enhanced induction of LTP at P28 in rats injected with BayK during the critical developmental periods of P7 and P14, we sought to determine whether a change in the expression of glutamatergic receptors in the BLA were associated with this effect. PCR from BayK-injected rats at P14 showed a decrease in the expression of GluA2AMPA receptor subunit. A decrease in GluA2 expression may suggest a shift in AMPA receptors to GluA2 lacking AMPA receptors, which are calcium permeable and capable of inducing LTP (Jia et al., 1996). Accordingly, the enhanced LTP observed here could result from an additive contribution of GluA2 lacking AMPA receptors to LTP at LA-BLA synapses (Whitehead et al., 2013). Interestingly, a Cav1.3-dependent increase of calcium permeable AMPA receptors has been previously shown in the ventral tegmental area-nucleus accumbens circuit of cocaine exposed mice (Martínez-Rivera et al., 2017). AMPA receptor dysregulation has also been linked to the social deficits seen in mice models of ASD, without impacting repetitive behaviors (Kim et al., 2019), consistent with our behavioral data from rats injected with BayK at P14 (Fig. 4*A*).

Reduced NMDA receptor function has been implicated in the pathophysiology of neuropsychiatric diseases; for instance, a mouse model with decreased GluN1 expression exhibits behavioral traits associated with ASD (Gandal et al., 2012). In line with this, we found a reduction in NMDA receptor subunits GluN1 and GluN2B in rats injected with BayK at P14, consistent with the social deficits exhibited by these rats (Fig. 4*A*). Conversely, the enhanced synaptic plasticity observed in this group (Fig. 5*B*), might seem counterintuitive with a reduction in GluN1 as in the BLA NMDA receptors are largely assembled as heterotrimers containing GluN1/GluN2B/GluN2A with the GluN2B subunit being essential for LTP (Delaney et al., 2013). Thus, a reduction in GluN1 and GluN2B would be predicted to reduce the induction of LTP. Nonetheless, combined with our AMPA receptor subunit data, these results suggest that LTCC manipulation at P14 alters glutamatergic receptor subunit expression which impacts synaptic plasticity and social behaviors at maturation. Lastly, in contrast to P14, BayK-injected rats at P7 did not show changes in expression of glutamatergic receptor subunits. Yet, BayK treatment led to an enhancement in LTP induction. This suggests that although there is a similar outcome when LTCCs are acutely enhanced during development, the underlying mechanisms that trigger this likely depends on the stage of development.

The molecular mechanisms underlying plasticity of glutamatergic synaptic transmission in the BLA have been studied under conditions triggering anxiety-like behavioral responses and/or the acquisition and consolidation of fear memories (Manzanares et al., 2005; Ryan et al., 2018). Furthermore, it has been reported that activation of BLA triggered by behavioral training induces a transient increase in mRNA expression of IEGs *c-fos*, *Homer 1a*, and *arc* (Knapska et al., 2007; Imamura et al., 2011; Nonaka et al., 2014; Rajbhandari et al., 2016). In the valproic acid (VPA) model of autism, high levels of *Homer 1a* mRNA and protein expression in the BLA have been correlated with an impairment of social behavior and fear conditioning (Banerjee et al., 2016), effects evident in adult animals treated at the embryonic stage, consistent with a long-term manifestation of transcriptional and functional changes. *c-fos* IEG has been widely used as an indirect reporter of neuronal activity as its expression is triggered by calcium influx through activated NMDA receptors and voltage-sensitive LTCCs (Rajbhandari et al., 2016; Hudson, 2018), mRNA expression levels were examined immediately after intra-amygdala injection of BayK during the critical period of development.

Here, BayK produced a significant transient increase of *c-fos* at 3 h after injection compared with the DMSO control group. No significant changes were observed in the mRNA expression of *Homer1a* in any experimental group. Statistically significant transient changes in *Arc* expression (1–3 h) were found between BayK and control groups, although the overall level of *Arc* expression was comparatively low. The lack of a BayK-evoked response in *c-fos* expression at P14 is difficult to explain although one could speculate that transcriptional regulation of *c-fos* at this age is more sensitive to the pattern of stimulus provoking neuronal activation (Tyssowski et al., 2018).

Overall, our results support the notion that altering LTCC activity at certain time points (P7 and P14) within the critical period of postnatal BLA development can produce long-lasting effects in adolescent life. Enhancing Ca^2+^ influx though LTCCs to then affect firing rates may alter the cues and sequence of events that ordinarily drive BLA immature networks to fully develop. Such dysregulation could eventually modify synaptic strength of LA-BLA inputs and reshape patterns of behavior linked to the amygdala even after a relatively long-time interval following drug treatment. Further, if these Ca^2+^-dependent alterations occur after the critical period (P21) the mature BLA neural circuits are not affected.

The present study may aid future research aimed at discerning the extent and mechanistic nature of enhanced LTCC activity within the BLA circuitry. Recent optogenetic studies have shown that the encoding of specific behavioral tasks (e.g., retrieval of fear memories or social interactive behavior) requires the activation of distinct subpopulations of BLA neurons (Bocchio et al., 2017). As such, further input-specific approaches using genetic strategies and employing relevant postnatal developmental stages (as demonstrated here and in Zhang et al., (2020) are needed to examine distinct BLA neuron-circuit ensembles and to target specific subsets of neurons expressing LTTCs (DeNardo and Luo, 2017).

## Synthesis

Reviewing Editor: Katalin Toth, University of Ottawa Decisions are customarily a result of the Reviewing Editor and the peer reviewers coming together and discussing their recommendations until a consensus is reached. When revisions are invited, a fact-based synthesis statement explaining their decision and outlining what is needed to prepare a revision will be listed below. The following reviewer(s) agreed to reveal their identity: Kurt Beam.

This study addresses an interesting a clinically relevant question by investigating the effect of LTCC activation in the context of neuronal development. While reviewers found the data solid, they identified few key questions that the authors would need to address. As Rev.#1 noted, the timeline and the exact nature of the changes caused by BayK injection are unclear. Rev.#2 pointed out uncertainty over the extent of the exact site of action of BayK. These two major issues would need to be addressed experimentally in order to make data interpretation unambiguous.

In addition, both reviewers make several additional points that could help the authors to improve the manuscript. Please follow eNeuro guidelines on statistics on all figures.

Rev.#1.

The authors report that transient pharmacological activation of LTCCs in the BLA using BayK early in the life of rats results in long-term behavioral changes. The behavioral changes are accompanied by modulation of intrinsic excitability and an enhanced ability to undergo LTP in BLA neurons. Finally, the authors show that mRNA levels controlling the expression of glutamate receptors subunits is differentially affected by BayK treatment. While the subject is generally interesting, the data is relatively hard to interpret. Below are my specific comments.

1) Most of the results presented here rely on the use of BayK, an LTCC agonist. A rationale for the use of BayK injection is the presence of gain-of-function mutations in LTCCs which results in ASD, for example Timothy Syndrome. However, BayK injection results in a transient effect, most likely not recapitulating the long-term effects produced by the TS mutations. Consistent with this idea, the underlying changes in LTCC activity induced by BayK injection remain unclear. What is the effect on BLA neurons? How long does BayK injection affect neurons? By how much is Ca2+ flux through LTCC enhanced? Without an answer to these questions, it remains difficult to extrapolate how BayK injection has an effect later in life at the time points investigated.

2) Are there any immediate behavioral changes in response to BayK injection in the BLA, such as seizures?

3) The electrophysiological changes reported may result from cellular and circuit reorganizations following BayK administration (and likely transient hyperexcitability in the network). It is hard to parse out whether these effects result from transiently enhanced LTCC activity or increased electrophysiological activity.

4) The gain in competence for synaptic plasticity is an interesting observation that deserves further investigation. I am curious whether this ability of the synapse to undergo LTP depends on LTCC themselves, or if other mechanisms are involved. In a naïve slice, does acute BayK application allow the synapse to show LTP? The authors could address this comment by using pharmacological dissection of the mechanisms underlying LTP.

5) Data related to Figure 4, such as input resistance, resting Vm and FI curves are not shown. The authors should consider adding these results to the figure.

6) There is an attempt to link the electrophysiological results showing the gain of competence in LTP to the expression of glutamate receptors subunits. The authors mention that the ratio of GluN2A/2B regulates the threshold for synaptic plasticity (Yashiron and Philpot, 2008; Paoletti et al., 2013). The results presented here do not show such a change. The authors suggest that the increased presence of GluA2 may contribute to support the enhanced ability to undergo LTP. However, no such increase is observed when injections are observed at P7. Therefore, the role of GluA2 in the ability to undergo LTP at this synapse remains to be determined.

7) In several graphs, multiple statistical comparisons are performed with Student’s t-test. It is unclear if the p-values are corrected for multiple comparisons.

Rev. #2.

This manuscript presents very clear-cut results: that behavior and synaptic physiology of P28 rats is altered by injection of a Bay K8644 solution into the basolateral amygdala (BLA) at P7 or P14, but not at P21. It also shows that glutamate receptor expression at P28 is altered by BayK injection at P14. Because BayK potentiates the activation of L-type calcium channels, the results imply that such channels are important for the development of the neural circuitry important for aversion and reward behaviors.

Major Comments:

1. Overall, these results provide new insights into the role of neuronal L-type channels in brain development, and thus merit publication. However, I think the authors must consider the possibility that the injected BayK affects L-channels in a relatively large volume of the brain. In particular, 0.5 ul (0.5 mm3) of a 1 mM BayK solution in DMSO was injected into the the BLA. The authors examined brains one hour after injection with the BayK solution (which is yellow), or injections with methylene blue, to confirm that the injection actually occurred into the BLA. However, these experiments do not reveal the spatial extent of the brain regions in which the concentration of BayK was sufficient to potentiate L-channels, or the time duration that such a concentration might have been present. Of course, the actual spatial distribution, and its duration, is likely to be complex and affected by brain structure and clearance via the vascular system. Nonetheless, it is an issue of concern since work from other labs indicates that 1 uM BayK strongly potentiates L-channels, meaning that the injected BayK could have produced such a concentration in a volume as large as 500 mm3. Thus, I believe that it is incumbent on the authors to have a section of the Discussion that explicitly states that there is uncertainty about the exact site of action of the injected BayK.

2. Figures 3 and 4 use normalized EPSC’s to determine whether high frequency stimulation induces LTP in the BLA, and whether this induction of LTP is altered by injection of BayK at P7, P14 or P21. What is missing from this analysis is the absolute size of the average EPSC’s prior to the high frequency stimulation. The authors should provide this information for BayK-injected and DMSO-only injected animals.

Minor Comments:

The title of the legend to Fig. 3 is “Bilateral BayK injection at P7 and P14 induce LTP in BLA neurons at P28.” This needs to be rewritten because it has a subject-verb error and does not accurately describe the results which show that LTP occurred in BLA neurons of rats injected with BK at P7 and P14, but not in animals injected with BK at P21.

The title of the legend to Fig. 4 is “Changes in synaptic strength induced after BayK treatment at P7 or P14 without preceding behavioral tests.” This needs to be rewritten because the results show that LTP occurs in the animals injected with BayK without preceding behavioral tests.

In the legend to Fig. 1 it is stated that “A significant reduction in inward rectification is observed in neurons from animals treated at P14 compared to DMSO control group (B, left and middle traces).” It looks to me that the inward rectification is increased.

## Author Response

Synthesis Statement for Author (Required):

This study addresses an interesting a clinically relevant question by investigating the effect of LTCC activation in the context of neuronal development. While reviewers found the data solid, they identified few key questions that the authors would need to address. As Rev.#1 noted, the timeline and the exact nature of the changes caused by BayK injection are unclear. Rev.#2 pointed out uncertainty over the extent of the exact site of action of BayK. These two major issues would need to be addressed experimentally in order to make data interpretation unambiguous.

In addition, both reviewers make several additional points that could help the authors to improve the manuscript. Please follow eNeuro guidelines on statistics on all figures.

Rev.#1.

The authors report that transient pharmacological activation of LTCCs in the BLA using BayK early in the life of rats results in long-term behavioral changes. The behavioral changes are accompanied by modulation of intrinsic excitability and an enhanced ability to undergo LTP in BLA neurons. Finally, the authors show that mRNA levels controlling the expression of glutamate receptors subunits is differentially affected by BayK treatment. While the subject is generally interesting, the data is relatively hard to interpret. Below are my specific comments.

1) Most of the results presented here rely on the use of BayK, an LTCC agonist. A rationale for the use of BayK injection is the presence of gain-of-function mutations in LTCCs which results in ASD, for example Timothy Syndrome. However, BayK injection results in a transient effect, most likely not recapitulating the long-term effects produced by the TS mutations. Consistent with this idea, the underlying changes in LTCC activity induced by BayK injection remain unclear. What is the effect on BLA neurons? How long does BayK injection affect neurons? By how much is Ca2+ flux through LTCC enhanced? Without an answer to these questions, it remains difficult to extrapolate how BayK injection has an effect later in life at the time points investigated.

The reviewer brings up several important points, which we have separated accordingly in our response below.

The rationale for using a single injection and the effect of BayK on BLA neurons: The demonstration that TS mutations cause gain-of-function effects on L-type Ca2+ calcium channel (LTCC) currents using heterologous systems (Splawski et al 2004) drew the attention of many researchers in the field, in part due to similarities to the gating and kinetic changes elicited by BayK on L-type Ca2+ currents (Barrett & Tsien, 2008). Along these lines, subsequent papers suggested that the mode of action of BayK recapitulates the functional impact of TS mutations, and could represent an alternative tool for examining neuronal behavior under conditions of increased activity of L-type channels (Sicouri et al, 2007; Barret & Tsien, 2008). In these regards, we published an initial study using acute application of BayK on BLA slices across different stages of rat postnatal development (Zhang et al 2020). With perfusion of 2 M BayK a main finding was an irreversible (over ∼20 to 30 min) increase in BLA neuronal intrinsic excitability at P7 (increased firing frequency, plateau potential and spike-frequency adaptation) with no changes in evoked synaptic responses. In contrast for P21 slices, the major effect of BayK was to increase bust firing, together indicating that BLA neurons at different developmental stages respond distinctly to the enhancement of LTCC activity.

We acknowledge that a single application of BayK imposes limitations concerning the notion of exactly mimicking L-type channel gain-of-function effects due to a genetic alteration of L-type channels. Rather, our approach here was to model any BLA pathologies associated with upregulation of LTCCs in a broader sense. We feel that our findings are both interesting and relevant as they indicate that an acute increase in LTCC activity generates BLA circuit alterations whose consequences manifest days to weeks after the pharmacological intervention. As such, the current study provides valuable insight for future experiments including the design of specific optogenetic approaches towards examining the downstream mechanisms triggered by LTCC activation, as pointed out in the last paragraph of the discussion.

A literature search did not reveal information concerning the half-life of BayK in brain tissue, however, considering the diffusion of a drug of its molecular weight and low aqueous solubility, the area of infiltration is estimated to be approximately 1 mm from the site of injection (Myers, 1966).

By how much is Ca2+ flux through LTCC enhanced? Ca2+ imaging experiments in BLA neurons from P18-P21 rats showed that 2.5 M BayK potentiated by 113 + 67 % Ca2+ influx evoked by subthreshold depolarizations in proximal dendrites (Power & Sah, 2005). In our case, we cannot explicitly provide a value for the magnitude of increase in Ca2+ influx produced by BayK in the BLA. Our purpose was to use the well-established L-type agonist activity of BayK as a tool to magnify the L-type mediated influx of Ca2+ in order to examine functional consequences in the basolateral amygdala. This is a conventionally accepted strategy to study the role of L-type channels in regulating neuronal activity in different brain areas (Hetzenauer et al, 2006; Lee & Tepper 2007; Busquet et al 2008; Striessnig et al, 2015)

In pilot testing for this study, we examined the effects of BayK on both recombinant Cav1.2 LTCCs expressed in HEK293 cells and on native LTCCs in undifferentiated PC12 cells in order to obtain predictive information concerning BayK effects on kinetic and voltage-dependent actions of (see Figure 1, unpublished data).

To directly examine BayK-induced changes in calcium entry in BLA neurons, we performed voltage-clamp experiments in coronal slices from rat brain with the purpose of calculating the total charge transference. However, generally poor space clamp and overall seal instability precluded an accurate estimation of Ca2+ influx. As shown in figure 2, we managed to maintain an acceptable level of voltage control and series resistance compensation in one cell using 20 M BayK. Similar to that for HEK and PC12 cells, the whole cell LTCC current was increased approximately 2.5-fold over control (see Figure 2, unpublished data).

Overall, while we cannot provide an exact quantitative answer to the reviewer’s question, we are convinced that the strategy of stimulating the entry of calcium through neuronal LTCCs using BayK represents a valid approach towards study of associated functional consequences in BLA neurons and circuitry.

2) Are there any immediate behavioral changes in response to BayK injection in the BLA, such as seizures?

In order to identify any adverse effect of the pharmacological treatment as well as general welfare indicators, all animals in the present study were carefully monitored both during the injection recovery period and after being placed back with their mother and littermates. We did not observe any signs of seizures or other neurological symptoms with the bilateral BLA injection of either vehicle or BayK. Early studies in rodents have reported a number of behavioral changes including dystonia, tonic-clonic seizures, and repetitive self-biting, however this was using intracerebroventricular administration of high doses of BayK (Shelton et al, 1987; Jinnah et al., 2000; Kasim & Jinnah, 2003).

3) The electrophysiological changes reported may result from cellular and circuit reorganizations following BayK administration (and likely transient hyperexcitability in the network). It is hard to parse out whether these effects result from transiently enhanced LTCC activity or increased electrophysiological activity.

Indeed, the working hypothesis of our study was that the functional relevance of LTCCs in BLA development relies on the ability to trigger transient hyperexcitability (see Zhang et al., 2020), which in turn could cause cellular and circuit reorganizations; this has been emphasized throughout the paper. For example, in the second paragraph of the Discussion, we state:

"In the rat BLA, application of BayK to acute brain slices from animals in the P7 to P21

critical period of development resulted in age-dependent changes in firing properties,

consistent with a crucial role of LTCCs in regulating neuronal excitability (Zhang et al.,

2020). However, that application of BayK in vivo could trigger permanent alterations in

membrane properties of BLA principal neurons which could functionally impact

amygdala excitability or associated behaviors at subsequent life stages remained to be

explored.”

After characterizing the acute effects of the L-type agonist in vitro in a previous study, we asked if the changes in firing patterns of BLA neurons could have biological significance in regulating amygdala-mediated functions. It is worth mentioning that we did not find changes in short-term plasticity, despite the fact that spontaneous synaptic activity indicated an overall increase in the amygdala excitability. Since circuit remodeling during development has been correlated to activity-dependent regulation of cellular processes, the age-dependent effects of BayK on excitability gave rise to the hypothesis that enhanced circuit activity triggered by L-type channel would impact the amygdala circuit during development.

The amygdala has long been known to be involved in the processing of emotions, social behavior and fear learning, and several behavioral paradigms have been established to evaluate the outcome of pharmacologic and genetic interventions in rodents; therefore, we decided to use it as a model to study the potential long-term consequences of increasing L-type activity on synaptic plasticity and behavior.

4) The gain in competence for synaptic plasticity is an interesting observation that deserves further investigation. I am curious whether this ability of the synapse to undergo LTP depends on LTCC themselves, or if other mechanisms are involved. In a naïve slice, does acute BayK application allow the synapse to show LTP? The authors could address this comment by using pharmacological dissection of the mechanisms underlying LTP.

To address this concern, we performed additional LTP experiments in naïve animals (Figure 3, unpublished data). Slice recordings from P28 naïve animals showed that HFS does not induce LTP under control conditions and further that acute BayK application does not modify this response. This supports the idea that cellular events triggered by upregulation of LTCC at early development stages (P7 and P14) confers the ability of this synapse to undergo LTP in response to HFS at P28.

5) Data related to Figure 4, such as input resistance, resting Vm and FI curves are not shown. The authors should consider adding these results to the figure.

The comment seems to refer to Figure 1, where we show current-voltage and the firing frequency relationships. We considered that adding the requested results to this figure although deemed that it would appear too crowded. Therefore, we have addressed this concern by including the estimation plots of passive membrane properties and neuronal firing parameters in new Figure 2 and Figure 3.

6) There is an attempt to link the electrophysiological results showing the gain of competence in LTP to the expression of glutamate receptors subunits. The authors mention that the ratio of GluN2A/2B regulates the threshold for synaptic plasticity (Yashiron and Philpot, 2008; Paoletti et al., 2013). The results presented here do not show such a change. The authors suggest that the increased presence of GluA2 may contribute to support the enhanced ability to undergo LTP. However, no such increase is observed when injections are observed at P7. Therefore, the role of GluA2 in the ability to undergo LTP at this synapse remains to be determined.

The reviewer makes an important point and we agree that it remains to be determined how the changes in the expression of glutamate receptor subunits impact LTP. This section was included for the purpose of explaining the rationale of the PCR experiments, and providing a general context concerning the relationship between glutamate receptor isoforms expression and synaptic plasticity.

There are multiple potential explanations for the LTP induction observed in rats injected with BayK at P7 and P14, including changes in the expression, trafficking and regulation of glutamatergic receptors. Although we focused our efforts on expression, we acknowledge that regulatory phosphorylation might also participate (Christian et al, 2012; Bessieres et al, 2019). Consequently, our results provide a starting point for future studies aimed at the modulation of BLA synaptic plasticity by highlighting the role of increased LTCC activity as relevant potential player. The GluNA2/2B ratio is mentioned in the results section to introduce how NMDA receptor subunits could regulate the threshold for synaptic plasticity. We do not make any statements that this ratio is changed in either experimental group.

In order to make this issue clearer to the reader, in the results we have changed the text to highlight that this statement is introductory:

"The subunit composition of ionotropic glutamatergic receptors can influence synaptic plasticity. For instance, the AMPA receptor subunit GluA2 has been implicated in the induction of LTP (Jia et al., 1996; Whitehead et al., 2013). While for NMDA receptors, the ratio of GluN2A/2B in adults has been suggested to regulate the threshold for synaptic plasticity (Yashiro & Philpot, 2008; Paoletti et al., 2013).”

We suggest as a possible mechanism in the P14 injected group that a reduction in GluA2 expression, consequently an increase in GluA2 lacking AMPA receptors, might underlie the increased ability to undergo LTP in this treatment group. However, this change was not seen in rats injected at P7. Hence, we cannot make any conclusions for this age group. The last sentence in this section of the discussion highlights this point by stating (p.24, line 8):

"Lastly, in contrast to P14, BayK injected rats at P7 did not show changes in expression of glutamatergic receptor subunits. Yet, BayK treatment led to an enhancement in LTP induction. This suggests that although there is a similar outcome when LTCCs are acutely enhanced during development, the underlying mechanisms that trigger this likely depends on the stage of development.”.

To further avoid any potential confusion, we have changed the following sentence in the discussion:

"PCR from BayK-injected rats at P14 showed a decrease in the expression of GluA2AMPA receptor subunit. A decrease in GluA2 expression may suggest a shift in AMPA receptors to GluA2 lacking AMPA receptors, which are calcium permeable and capable of inducing LTP (Jia et al., 1996).”

7) In several graphs, multiple statistical comparisons are performed with Student’s t-test. It is unclear if the p-values are corrected for multiple comparisons.

The p-values only represent the significant differences between DMSO and BayK within its own age group and were not corrected for multiple comparisons. As suggested by eNeuro guidelines, we have included estimation analysis of electrophysiological data. This type of analysis provides more in-depth statistical information about precision of the measurements and quantitative answers about the magnitude of the effect (Bernard, 2019; Calin-Jageman and Cumming, 2019; Gardner and Altman, 1986; Ho et al., 2019) (new references have been added). Estimation analysis values are now reported following hypothesis testing values as follow: difference between mean [95% confidence interval (CI)]. Using this analysis, one statistically significant parameter was modified: input resistance for rats injected at P14 (previously significant p<0.05, and now as follows: DMSO 77.1{plus minus}11.3 MΩ, n=5, BayK 128.9{plus minus}20.6 MΩ, n=11, p=0.128 mean difference 51.79 [95% CI -16.87 to 120.4]). We have modified the text and included new figures accordingly (Figure 2 and Figure 3).

Rev. #2.

This manuscript presents very clear-cut results: that behavior and synaptic physiology of P28 rats is altered by injection of a Bay K8644 solution into the basolateral amygdala (BLA) at P7 or P14, but not at P21. It also shows that glutamate receptor expression at P28 is altered by BayK injection at P14. Because BayK potentiates the activation of L-type calcium channels, the results imply that such channels are important for the development of the neural circuitry important for aversion and reward behaviors.

Major Comments:

1. Overall, these results provide new insights into the role of neuronal L-type channels in brain development, and thus merit publication. However, I think the authors must consider the possibility that the injected BayK affects L-channels in a relatively large volume of the brain. In particular, 0.5 μl (0.5 mm3) of a 1 mM BayK solution in DMSO was injected into the the BLA. The authors examined brains one hour after injection with the BayK solution (which is yellow), or injections with methylene blue, to confirm that the injection actually occurred into the BLA. However, these experiments do not reveal the spatial extent of the brain regions in which the concentration of BayK was sufficient to potentiate L-channels, or the time duration that such a concentration might have been present. Of course, the actual spatial distribution, and its duration, is likely to be complex and affected by brain structure and clearance via the vascular system. Nonetheless, it is an issue of concern since work from other labs indicates that 1 μM BayK strongly potentiates L-channels, meaning that the injected BayK could have produced such a concentration in a volume as large as 500 mm3. Thus, I believe that it is incumbent on the authors to have a section of the Discussion that explicitly states that there is uncertainty about the exact site of action of the injected BayK.

The reviewer points out an important issue that we have better described in the revised methods section. Based on the changing amygdala volume throughout development in Sprague-Dawley rats (Calabrese et al. 2013), we estimate that injecting 0.5μl of a 1 mM stock solution of BayK into the amygdala would result in a range of concentrations between 12 μM -18 μM in P7 to P21 animals. Although this seems somewhat high, it is equivalent to 8 μg/kg, which is significantly lower than previously reported for intracerebroventricular injection (5-20 μg/kg) (Bourson et al., 1989) or intraperitoneal injection (2-4 mg/kg) (Sinnegger-Brauns et al., 2004). Based on the molecular weight of BayK (Myers, 1966) and considering the low aqueous solubility, the solution injected is predicted to spread no more than 1 mm from the site of injection. This is consistent with our observations using methylene blue showing that after 1 hour, 0.5μl of dye dissolved in saline solution covered a total diameter of ∼1 mm of the BLA, without extending beyond the external capsule.

2. Figures 3 and 4 use normalized EPSC’s to determine whether high frequency stimulation induces LTP in the BLA, and whether this induction of LTP is altered by injection of BayK at P7, P14 or P21. What is missing from this analysis is the absolute size of the average EPSC’s prior to the high frequency stimulation. The authors should provide this information for BayK-injected and DMSO-only injected animals.

The data of absolute size of the average EPSCs has been added to the text in the LTP results section and with the relevant Figures now Figure 5 and Figure 6.

Minor Comments:

a) The title of the legend to Fig. 3 is “Bilateral BayK injection at P7 and P14 induce LTP in BLA neurons at P28.” This needs to be rewritten because it has a subject-verb error and does not accurately describe the results which show that LTP occurred in BLA neurons of rats injected with BK at P7 and P14, but not in animals injected with BK at P21.

The title (now figure 5) has been changed to “LTP was induced in P28 BLA neurons that received bilateral BayK injection at P7 and P14, but not at P21.”

b) The title of the legend to Fig. 4 is “Changes in synaptic strength induced after BayK treatment at P7 or P14 without preceding behavioral tests.” This needs to be rewritten because the results show that LTP occurs in the animals injected with BayK without preceding behavioral tests.

Upper panels of Figure 4 (now Figure 6) show the time course of the LTP induction in two groups of animals which have not been exposed to behavioral experience and which we tried to highlight in the title. However, the lower panels show the steady state EPSCs ratios obtained from the group of animals previously tested for behavior, compared to the values from animals without behavioral experience. We agree with the reviewer that the title might create confusion; we have changed the title to:

Figure 6. Changes in synaptic strength induced after BayK treatment at P7 or P14 occur without preceding behavioral tests.

c) In the legend to Fig. 1 it is stated that “A significant reduction in inward rectification is observed in neurons from animals treated at P14 compared to DMSO control group (B, left and middle traces).” It looks to me that the inward rectification is increased.

In the DMSO experimental group, inward rectification can be observed as a small decrease in slope resistance with hyperpolarizing pulses that bring membrane potential approximately 5 to 10 mV negative to the Vr. It is true that at first glance, the I-V curve from BayK treated BLA neurons appears non-linear, giving the impression of an increase in rectification. However, changes in the hyperpolarizing voltage responses at P14 seemed more complex; therefore, we decided to calculate the rectification ratio as well as the sag index in order to provide a more precise description of the BayK effect. Changes in the manuscript were made to address this concern in page 11-12.

-

REFERENCES

Bernard C (2019) Changing the way we report, interpret, and discuss our results to rebuild trust in our research. eNeuro, 6:ENEURO.0259-19.2019.

Barrett CF, Tsien RW (2008). The Timothy syndrome mutation differentially affects voltage- and calcium-dependent inactivation of CaV1.2 L-type calcium channels. Proc Natl Acad Sci U S A 105(6):2157-62.

Bessières B, Jia M, Travaglia A, Alberini CM (2019) Developmental changes in plasticity, synaptic, glia, and connectivity protein levels in rat basolateral amygdala. Learn Mem 26:436-448.

Bourson, A., Moser, P. C., Gower, A. J., & Mir, A. K. (1989). Central and peripheral effects of the dihydropyridine calcium channel activator BAY K 8644 in the rat. European journal of pharmacology, 160(3), 339-347.

Calin-Jageman RJ & Cumming G (2019). Estimation for better inference in Neuroscience. eNeuro 6, ENEURO.0205-19.2019.

Calabrese E, Badea A, Watson C, Johnson GA (2013) A quantitative magnetic resonance histology atlas of postnatal rat brain development with regional estimates of growth and variability. Neuroimage 71:196-206.

Christian DT, Alexander NJ, Diaz MR, Robinson S, McCool BA (2012) Chronic intermittent ethanol and withdrawal differentially modulate basolateral amygdala AMPA-type glutamate receptor function and trafficking. Neuropharmacology 62:2430-2439.

Farries MA, Meitzen J, Perkel DJ. (2005) Electrophysiological properties of neurons in the basal ganglia of the domestic chick: conservation and divergence in the evolution of the avian basal ganglia. J Neurophysiol. 94(1):454-67

Gardner MJ & Altman DG (1986). Confidence intervals rather than P values: estimation rather than hypothesis testing. Br Med J Clin Res Ed 292, 746-750.

Ho J, Tumkaya T, Aryal S, Choi H & Claridge-Chang A (2019). Moving beyond P values: data analysis with estimation graphics. Nat Methods 16, 565-566.

Hetzenauer A, Sinnegger-Brauns MJ, Striessnig J, Singewald N (2006). Brain activation pattern induced by stimulation of L-type Ca2+-channels: contribution of Ca(V)1.3 and Ca(V)1.2 isoforms. Neuroscience. 139(3):1005-15.

Jarsky T, Mady R, Kennedy B, Spruston N. (2008) Distribution of bursting neurons in the CA1 region and the subiculum of the rat hippocampus. J Comp Neurol. 506(4):535-47.

Jinnah HA, Sepkuty JP, Ho T, Yitta S, Drew T, Rothstein JD, Hess EJ. (2000). Calcium channel agonists and dystonia in the mouse. Mov Disord. 15(3):542-51.

Kasim S, Jinnah HA. (2003). Self-biting induced by activation of L-type calcium channels in mice: dopaminergic influences. Dev Neurosci. 25(1):20-5.

Lee, C. R., & Tepper, J. M. (2007). A calcium-activated nonselective cation conductance underlies the plateau potential in rat substantia nigra GABAergic neurons. J Neurosci. 27(24), 6531-6541.

Myers R.D. (1966). Injection of Solution into cerebral tissue: relation between volume and diffusion. Physiology and Behavior 1: 171-174

Splawski I, Timothy KW, Sharpe LM, Decher N, Kumar P, Bloise R, Napolitano C, Schwartz PJ, Joseph RM, Condouris K, Tager-Flusberg H, Priori SG, Sanguinetti MC, Keating MT. (2004) Ca(V)1.2 calcium channel dysfunction causes a multisystem disorder including arrhythmia and autism. Cell. 119(1):19-31

Sicouri, S., Timothy, K. W., Zygmunt, A. C., Glass, A., Goodrow, R. J., Belardinelli, L., & Antzelevitch, C. (2007). Cellular basis for the electrocardiographic and arrhythmic manifestations of Timothy syndrome: effects of ranolazine. Heart rhythm, 4(5), 638-647.

Shelton RC, Grebb JA, Freed WJ. (1987). Induction of seizures in mice by intracerebroventricular administration of the calcium channel agonist BAY k 8644. Brain Res. 402(2):399-402.

Striessnig J, Ortner NJ, Pinggera A. (2015). Pharmacology of L-type Calcium Channels: Novel Drugs for Old Targets? Curr Mol Pharmacol. 8(2):110-22.

Sinnegger-Brauns, M. J., Hetzenauer, A., Huber, I. G., Renström, E., Wietzorrek, G., Berjukov, S., Cavalli, M., Walter, D., Koschak, A., Waldschütz, R., Hering, S., Bova, S., Rorsman, P., Pongs, O., Singewald, N., & Striessnig, J. (2004). Isoform-specific regulation of mood behavior and pancreatic beta cell and cardiovascular function by L-type Ca 2+ channels. The Journal of clinical investigation, 113(10), 1430-1439.

Zhang Y, Garcia E, Sack AS, Snutch TP. (2020). L-type calcium channel contributions to intrinsic excitability and synaptic activity during basolateral amygdala postnatal development. J Neurophysiol. 123(3):1216-1235.
